# Understanding the Electron Beam Resilience of Two-Dimensional Conjugated Metal–Organic Frameworks

**DOI:** 10.1021/acs.nanolett.3c04125

**Published:** 2024-03-01

**Authors:** David Mücke, Isabel Cooley, Baokun Liang, Zhiyong Wang, SangWook Park, Renhao Dong, Xinliang Feng, Haoyuan Qi, Elena Besley, Ute Kaiser

**Affiliations:** † Central Facility for Materials Science Electron Microscopy, Universität Ulm, 89081 Ulm, Germany; ‡ School of Chemistry, University of Nottingham, University Park, Nottingham NG7 2RD, United Kingdom; § Max Planck Institute of Microstructure Physics, 06120 Halle (Saale), Germany; ∥ Faculty of Chemistry and Food Chemistry & Center for Advancing Electronics Dresden, Technische Universität Dresden, 01062 Dresden, Germany; ⊥ Key Laboratory of Colloid and Interface Chemistry of the Ministry of Education, School of Chemistry and Chemical Engineering, Shandong University, 250100 Jinan, China

**Keywords:** beam damage, metal organic frameworks, high-resolution transmission electron microscopy, structural tailoring, ab initio molecular dynamics

## Abstract

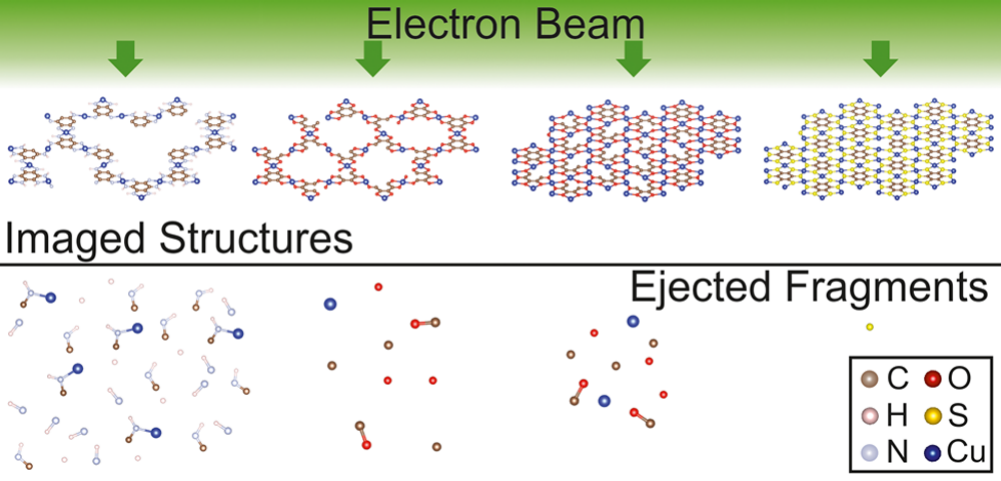

Knowledge of the atomic structure of layer-stacked two-dimensional conjugated metal–organic frameworks (2D c-MOFs) is an essential prerequisite for establishing their structure–property correlation. For this, atomic resolution imaging is often the method of choice. In this paper, we gain a better understanding of the main properties contributing to the electron beam resilience and the achievable resolution in the high-resolution TEM images of 2D c-MOFs, which include chemical composition, density, and conductivity of the c-MOF structures. As a result, sub-angstrom resolution of 0.95 Å has been achieved for the most stable 2D c-MOF of the considered structures, Cu_3_(BHT) (BHT = benzenehexathiol), at an accelerating voltage of 80 kV in a spherical and chromatic aberration-corrected TEM. Complex damage mechanisms induced in Cu_3_(BHT) by the elastic interactions with the e-beam have been explained using detailed *ab initio* molecular dynamics calculations. Experimental and calculated knock-on damage thresholds are in good agreement.

Recent years have witnessed the rise in applications of two-dimensional layer-stacked conjugated metal–organic frameworks (2D c-MOFs) making effective use of their intrinsic conductivity, anisotropic charge transport, and (opto-)electronic properties.^1^ Tremendous efforts have been devoted to band structure engineering of these emerging materials, which exhibit strong in-plane π-*d* conjugation, using the controlled assembly of transition metal nodes and aromatic ligands. This represents a challenging endeavor on synthetic and characterization frontiers with interfacial synthesis emerging as a new paradigm for the production of highly crystalline 2D c-MOFs.^1−3^

Understanding the rational correlation between the electronic structure of 2D c-MOFs and the underlying atomic structure remains a formidable task that can be achieved through a combination of computational chemistry and imaging of these layer-stacked 2D structures with atomic resolution. However, in aberration-corrected high-resolution transmission electron microscopy (AC-HRTEM), electron radiation damage, *i.e*. atomic displacement, bond scission, and chemical etching, can lead to instantaneous amorphization of organic materials during imaging,^4,5^ severely limiting the achievable resolution. Additionally, as the thickness of the sample decreases from bulk toward the monolayer limit, the mechanism of the damage process needs to be revisited. Operating the AC-HRTEM in low-dose mode can be important to extract high-resolution information with a minimum illumination.^6−8^ Direct electron detectors have triggered a resolution revolution due to their exceptional detective quantum efficiency,^9^ for example, enabling sub-2 Å information transfer on UiO-66 MOF with 12 e^–^/Å^2^.^6^ Progress was also made in the application of advanced imaging techniques like iDPC-STEM^10^ and electron ptychography.^11^ Despite the remarkable advances in instrumentation, the intrinsic sensitivity of MOFs to the electron beam severely restricts the dose budget for high-resolution information. For instance, an ultralow dose of 4.1 e^–^/Å^2^ is required to retain 2.1 Å resolution in ZIF-8.^7^

In this work, we investigate four selected 2D c-MOFs consisting of Cu and benzene-ring-based organic ligands designed with altered structural attributes to study their resilience to the electron beam in the TEM experiment. Specifically, we study how stability under the electron beam is influenced by the structural features of the four 2D c-MOF structures, shown in Figure 1, labeled Cu_3_(HIB)_2_, Cu_3_(HHB)_2_, Cu_3_(HHB), and Cu_3_(BHT) (HIB = hexaiminobezene, HHB = hexahydroxybenzene, and BHT = benzenehexathiol). We aim to shed light on the sample-dependent parameter field for resolution enhancement. We focus primarily on the intrinsic properties of 2D c-MOFs which can affect the e-beam resilience of these materials such as hydrogen content, porosity, and electrical conductivity. For Cu_3_(BHT) 2D c-MOF, we perform for the first time a comparative study of the damage mechanisms from *ab initio* molecular dynamics and C_C_/C_S_ corrected 80 kV HRTEM imaging.

**Figure 1:**
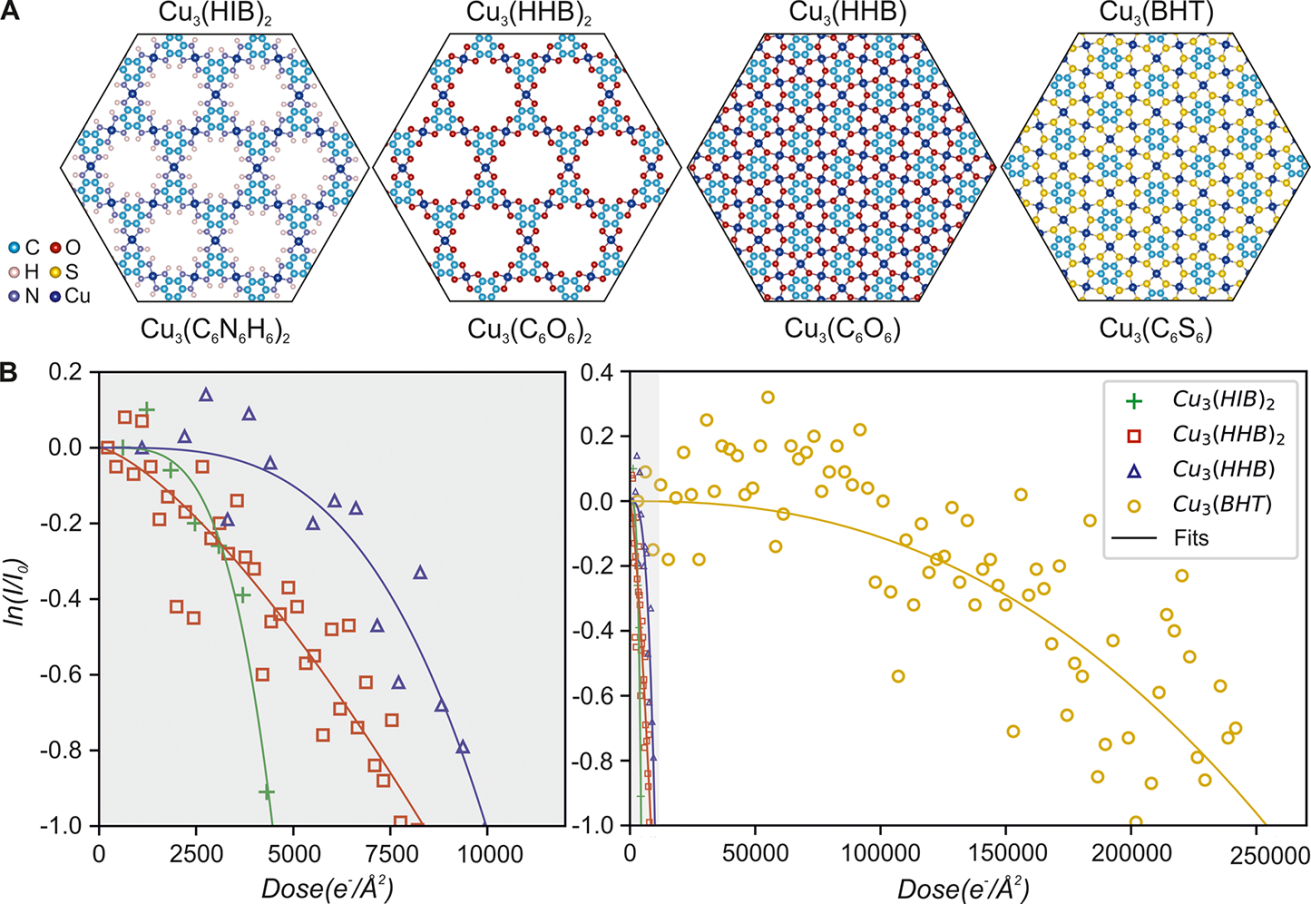
Atomic structures and electron resilience of 2D c-MOFs. (A) Atomic models of the 2D c-MOFs. Cu_3_(HIB)_2_ and Cu_3_(HHB)_2_ exhibit a porous honeycomb lattice, whereas Cu_3_(HHB) and Cu_3_(BHT) adopt a nonporous hexagonal framework structure. (B) Intensity profile of the first-order reflections in 2D c-MOFs as a function of accumulated electron dose. The critical dose is reached when the reflection intensity drops to *e*^–1^ of the original value. Adapted with permission under a Creative Commons CC BY 4.0 from ref (18). Copyright 2023 Baokun Liang.

## Results and Analysis

For inorganic 2D materials, AC-HRTEM has demonstrated remarkable success in acquiring sub-angstrom resolution images,^12,13^ and many experiments and calculations have been devoted to the quantitative determination of the damage cross section.^14,15^ High-quality monolayers of inorganic 2D materials are readily available, and their stability is high enough to acquire images with atomic resolution and to directly measure atomic positions with picometer precision, even in a defective crystal. Theoretical insights have been particularly helpful in further analysis of the damage process of inorganic 2D materials;^16,17^ however, this level of understanding of damage mechanisms in 2D c-MOFs is still lacking in the literature.

In a TEM experiment, sample damage is best analyzed by the vanishing of diffraction spots in the electron diffraction patterns. However, due to the limited size of single crystalline domains of the 2D c-MOFs in this study, it is difficult to obtain electron diffraction patterns of a single crystalline domain with a sufficient signal. For this reason, the power spectrum of the AC-HRTEM images was used to assess the crystallinity of the samples.

### Structures of the 2D c-MOFs

Figure 1A presents the atomic structures of the 2D c-MOFs selected for this work. Both Cu_3_(HIB)_2_ and Cu_3_(HHB)_2_ consist of a porous honeycomb framework with comparable lattice parameters. The coordination reaction between 1,2,3,4,5,6-hexahydroxybenzene and Cu ions results in the absence of hydrogen atoms in Cu_3_(HHB)_2_, whereas N–H bonds are present at the pore interface of Cu_3_(HIB)_2_. The HHB ligands can also polymerize into a nonporous hexagonal lattice, i.e., Cu_3_(HHB), doubling the Cu node density compared to its porous counterpart. Cu_3_(BHT) differs from Cu_3_(HHB) in organometallic bonding.

The lattice parameters, chemical formulas, and reported conductivity are summarized in Table 1. Note that metal nodes in 2D MOFs could convert absorbed ionization energy into fast electron emission,^19^ an effect dependent on the atomic number. Therefore, we consider only Cu nodes to avoid potential metal dependency on ionization damage. In addition, the thickness of all 2D c-MOFs lies in a narrow range, *i.e*. from a few to a few tens of nanometers (see Figure S1 in Supporting Information), which suppresses the thickness effect on radiolysis induced by secondary electrons.^20^ To quantify the electron resilience, image series with accumulated electron dose have been acquired at an acceleration voltage of 300 kV (see Figure S2 and Methods Section in Supporting Information).

**Table 1:** Critical Dose for Total Amorphization, Chemical Formula, Symmetry, Lattice Parameters, and Electrical Conductivity of the Four 2D c-MOFs

HIB: hexaiminobenzene; HHB: hexahydoxybenzene; BHT: benzenehexathiol					
2D MOFs	Chemical formula	Symmetry	Lattice parameters (Å)	Electrical conductivity at 298 K (S/cm)	Critical dose (e^–^/Å^2^)
Cu_3_(HIB)_2_	Cu_3_(C_6_N_6_H_6_)_2_	honeycomb	*a* = 13.5	13	(4.45 ± 0.31) × 10^3^
			*c* = 3.3		
Cu_3_(HHB)_2_	Cu_3_(C_6_O_6_)_2_	honeycomb	*a* = 13.1	7.3 × 10^–8^	(7.80 ± 0.89) × 10^3^
			*c* = 3.0		
Cu_3_(HHB)	Cu_3_(C_6_O_6_)	hexagonal	*a* = 7.5	2.6 × 10^–2^	(8.88 ± 0.91) × 10^3^
			*c* = 2.9		
Cu_3_(BHT)	Cu_3_(C_6_S_6_)	hexagonal	*a* = 8.5	2500	(2.48 ± 0.77) × 10^5^
			*c* = 3.5		

### Stability under the Electron Beam

The critical electron dose for total amorphization *D*_*a*_ is reached when the intensity of first order reflections in the power spectrum drops to *e*^–1^ of the initial value. Figure 1B shows the intensity profiles of the first-order reflections ⟨101̅0⟩ as a function of the accumulated dose, and the values of *D*_*a*_ of the 2D c-MOFs are listed in Table 1. During image series acquisition, the pre-specimen shutter was used to avoid illumination during camera read out periods. Since some thermal and time-dependent damage processes are influenced by the applied dose rate,^21^ the same dose rate of 1200 e^–^/Å^2^s was used for AC-HRTEM imaging of all structures to prevent the effects of using different dose rates.

### 300 kV Cs-Corrected Low-Dose HRTEM Imaging

The resolution obtained in the high-resolution TEM image of a specimen under investigation (in brief, the specimen resolution *d*_s_) is strongly dependent on the critical dose the specimen can accept before it is destroyed:^22,23^
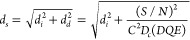
1where, *d*_*i*_ is the resolution of the instrument, *d*_*d*_ is the dose-limited resolution, *S*/*N* is the signal-to-noise ratio, *C* is the image contrast, *D*_*c*_ is the critical dose, and *DQE* stands for detective quantum efficiency of the camera. To detect an unknown signal in the presence of noise, typically, an *S*/*N* of 4 to 5 is required.^24^ Thus, using structures that can sustain the highest critical dose is a prerequisite to achieving the highest image resolution.

**Figure 2:**
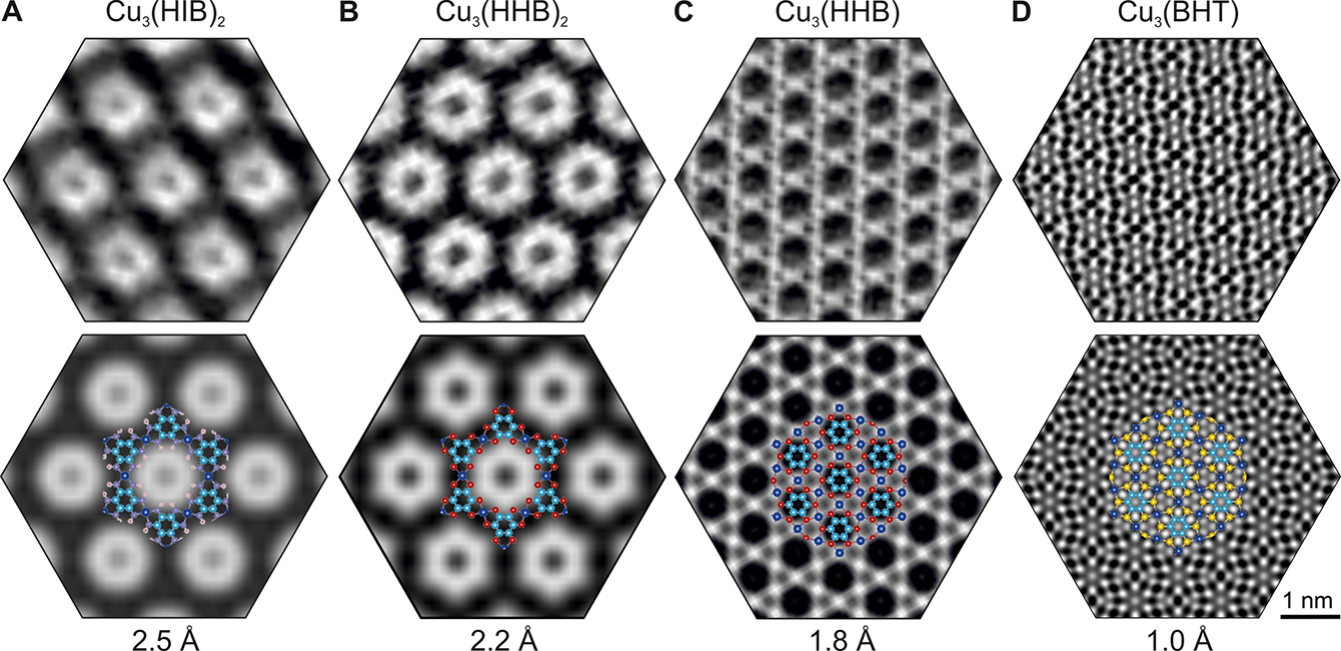
300 kV *C*_s_-corrected HRTEM of 2D c-MOFs. Upper row: experimental HRTEM images. Unit-cell real-space averaging has been applied to enhance the S/N ratio (Methods). Lower row: simulated HRTEM images with atomic models overlaid. The achieved resolution in the unprocessed images is specified. Image acquisition dose: A: 100 e^–^Å^–2^, B–C: 200 e^–^Å^–2^; D: 5.6 × 10^3^ e^–^Å^–2^. Adapted with permission under a Creative Commons CC BY 4.0 from ref (18). Copyright 2023 Baokun Liang.

Tailoring the structure led to an improvement in the acceptable critical dose during imaging. To demonstrate this improvement, we acquired AC-HRTEM images of each sample at 300 kV (Figure 2). For Cu_3_(HIB)_2_, a resolution of 2.5 Å was achieved with ca. 100 e^–^/Å^2^ and the experimental image agrees well with the simulated image, as shown in Figure 2A (also, Figure S3) with unit-cell real-space averaged (see Methods, Supporting Information) images. The electron dose was optimized to balance high-resolution information and signal-to-noise ratio.^25^ The hydrogen-free Cu_3_(HHB)_2_ exhibits a 2-fold increase in the damage cross section *D*_*a*_ as compared to Cu_3_(HIB)_2_ (see Figure 1B, Table 1). The higher stability of Cu_3_(HHB)_2_ translates into an improvement in achievable image resolution, reaching 2.2 Å (Figure 2B, also, Figure S4 in Supporting Information). Nonetheless, the longest bond length, *i.e*., Cu–O (2.0 Å), lies below the achieved resolution. Although the molecular framework could be unambiguously visualized, no atomic columns were resolved. The nonporous counterpart of the porous HHB-based 2D c-MOF, *i.e*. Cu_3_(HHB), displays a further improved radiation stability (Figure 1B). In this case, the information transfer extends to 1.8 Å, resolving the Cu–O kagome lattice with trihexagonal tiling (Figure 2C). Under the experimental defocus value, contrast reversal occurs at benzene rings (Figure S5 in Supporting Information). However, with contrast transfer function correction (Figure S8 in Supporting Information), no information could be retrieved at carbon sites, suggesting insufficient signal from carbon atoms at an acquisition dose limited to 200 e^–^ /Å^2^.

The exceptional electron resilience endows Cu_3_(BHT) with a substantially increased tolerable acquisition dose. Figure 2D presents the AC-HRTEM image of Cu_3_(BHT) with an image resolution of 1.0 Å (Figure S6 in Supporting Information). Since the shortest bond length in Cu_3_(BHT) amounts to 1.4 Å (C–C bond, see Figure S9 in Supporting Information), Cu, S, and benzene rings have been all unambiguously resolved.

### Understanding the Electron Stability of the c-MOFs

To explain why the thin specimens of the studied structurally modified 2D c-MOFs show different stability under the e-beam, we suggest three main reasons: *First*, as expected, the replacement of the hydrogen–nitrogen fragments with oxygen increased the stability of the framework notably, *by a factor of 2*. This is due to the fact that hydrogen-containing bonds are weaker and get destroyed rapidly by the e-beam.^26^ Also, once the hydrogen atoms are ejected from the structure, the associated nitrogen atoms become destabilized. *Second*, altering the density of copper centers in 2D c-MOF structures leads to a small difference in the critical dose. In the dense 2D c-MOF structures, the percentage of the damage with respect to the whole area remains the same as in the porous structure (two ejected atoms in the dense lattice result in the same percent damage as one atom in the porous lattice). The increase in atomic density may enhance the cage effect whereby knocked out fragments are hindered and are unable to escape the sample leading to recombination and self-healing of the lattice.^27^ In the dense material, the fragments’ escape can be prevented. In the porous counterpart, however, channels are formed in the thicker crystal, and knocked-out fragments can escape through these channels leading to a lower recombination rate and higher damage rate.^28^*Finally*, the biggest structure stabilization effect has been achieved by replacing the oxygen atoms with sulfur. Through this chemical substitutional change, the critical dose was increased nearly *by a factor of 30*, setting the stability of the Cu_3_(BHT) c-MOF far above the other 2D structures. This enormous change in the stability of Cu_3_(BHT) points to a fundamental difference in its physical properties.

Changing the structure of c-MOFs not only alters the chemical bonding but also can alter other properties that affect the stability of a c-MOF in the e-beam. For the Cu_3_(BHT) c-MOF, the electrical conductivity plays a major role. Samples with higher conductivity have a greater potential to replace missing electrons or drain excess electron density, and the excited electrons have a faster relaxation rate; all of these processes significantly reduce radiolysis damage to the sample. Cu_3_(HIB)_2_ has a conductivity of 13 S cm^–1^^29^ which is much higher compared to that of Cu_3_(HHB)_2_,^30^ and Cu_3_(HHB) (unpublished data) structures (see Table 1). However, due to the high hydrogen content, Cu_3_(HIB)_2_ is destroyed very quickly during imaging, and the conductivity of the material cannot compensate for the rapid destruction. The stability of Cu_3_(HIB)_2_ is comparable to the stability of the copper phthalocyanine (CuPc) self-assembly, which is a hydrogen-containing molecule, and the thin film of CuPc has the same order of conductivity as Cu_3_(HIB)_2_.^31^ As one of the most stable organic molecules, the dominating damage via displacement and radiolysis of bonds containing hydrogen leads to more than 1 order of magnitude lower stability than previous prediction.^32^

A huge difference, however, was found for the Cu_3_(BHT) structure. Due to the strong π-*d* interactions and electron delocalization,^33^ Cu_3_(BHT) exhibits an electrical conductivity of 2500 S cm^–1^, which is by far the highest among 2D c-MOFs.^34^ At the present stage, the conductivity of the Cu_3_(BHT) is measured on a thin film, which might be affected by domain boundaries and defects.^35^ In the TEM measurements of single crystalline domains, the electron conductivity could be even higher than the reported value. For typical c-MOFs, radiolysis is by far the largest contributor to e-beam damage but not for the H-free Cu_3_(BHT). Here, the high conductivity suppresses radiolysis effects, and the remaining damage mechanism is knock-on damage.

### Knock-on Damage of Cu_3_(BHT)

**Figure 3:**
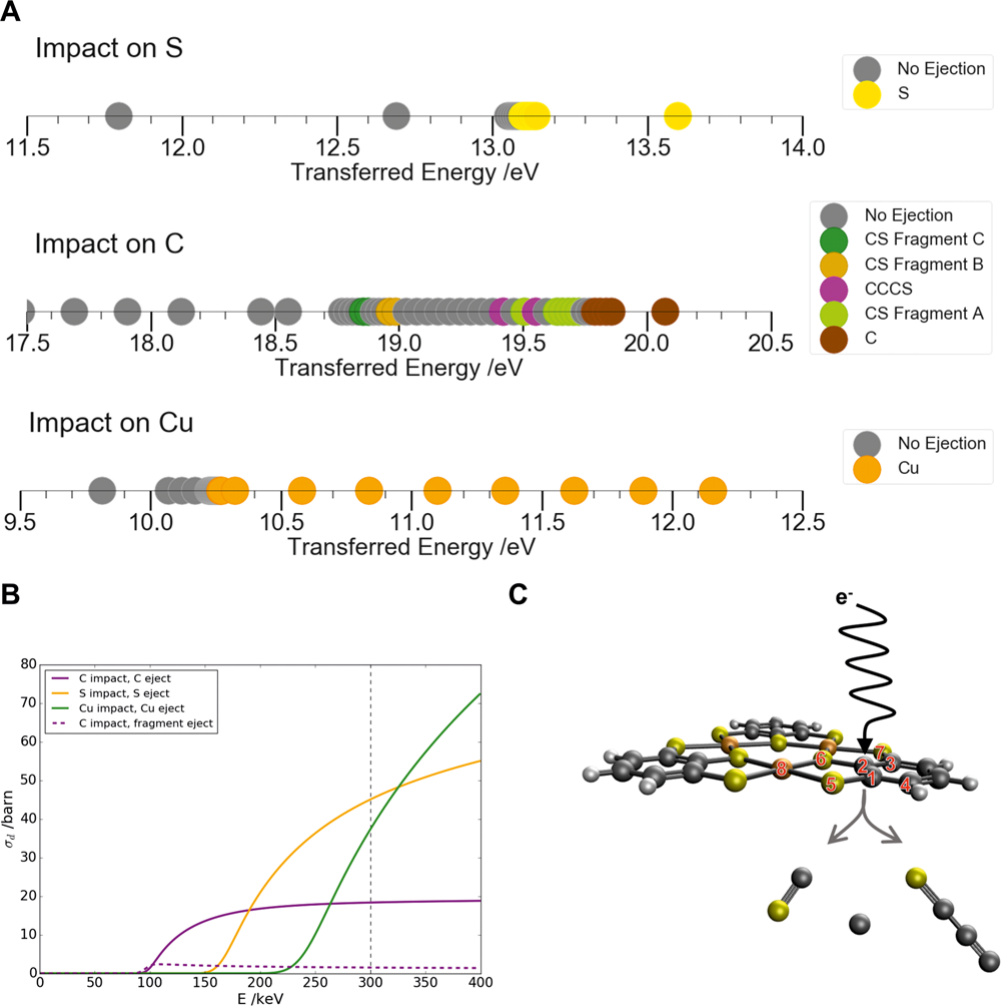
*Ab initio* dynamics (AIMD) results for the ejection thresholds and cross sections. (A) Number lines show fragmentation events over a range of transferred energies. (B) Knock-on cross sections following impacts on each atom type, split by fragmentation event. (C) Diagram of ejections following impacts on C, with all atoms involved in ejection events labeled numerically. Using the numbering system established in (C), the ejected atoms and fragments are identified as follows. S: S6; C: C2; Cu: Cu8; CCCS: C2C4C1S5; CS fragment A: C1S5; CS fragment B: C2S5; CS fragment C: C3S7.

Due to the dominance of knock-on damage, the interaction between the e-beam and Cu_3_(BHT) is similar to that of inorganic 2D materials such as graphene and transition metal dichalcogenides (TMDs).^14,15^ Computational analysis of the knock-on processes^16,17^ can be applied to describe the sample damage. In doing so, a vast amount of different intricate ejection processes has been detected in the Cu_3_(BHT) 2D c-MOF due to its structural complexity. To break down the damage cross section, we consider the computational cross section by primary knock-on atom (PKA) and by fragmentation energy; these *ab initio* molecular dynamics calculations have been done on a small Cu_3_(BHT) fragment as depicted in Figure 3C. Following a knock-on event, *i.e*. an electron hitting the PKA, different ejection processes have been identified, which depend on the energy transferred from an incident electron to the PKA. If the transferred energy is too small, no ejection occurs. As the transferred energy increases, the probability of ejecting a fragment goes up, and above a certain threshold, the primary ejection takes place in which the PKA is directly ejected on its own. Between these two limiting cases, the relaxation of the PKA during a knock-on event can cause sufficient structural disturbance to the sample for the process to be followed by the ejection of small fragments. The resulting intermediate fragmentation pathways consist of various small molecular particulates, which often include the PKA.

In Figure 3A, these different fragmentation pathways are summarized to show that several intermediate fragmentation pathways contribute to knock-on damage to carbon atoms, for example. These pathways range from the ejection of C–S fragments to larger fragments consisting of three carbon and one sulfur atom. On the other hand, knock-on damage to heavier atoms, like sulfur and copper, involves only the primary ejection pathway. In Figure 3B, the ejection cross section of each atom is plotted with respect to the acceleration voltage ranging from 0 to 400 kV. The observed prevalence of intermediate fragmentation pathways for damage to C atoms, their complex changing nature with the transferred energy, and the presence of nonejection events within the energy range that they cover have implications for modeling of damage events. They indicate that to fully describe damage, it is sufficient neither to identify the threshold for primary ejection nor to identify the lowest threshold for ejection of any fragment. Further examples of this phenomenon and its potential to significantly influence total damage are illustrated in the Supporting Information using *ab initio* dynamics simulations relevant to the remaining three c-MOFs.

### 80 kV C_c_/C_s_ Corrected Low-Dose HRTEM Imaging

The outstanding stability of the Cu_3_(BHT) sample encouraged imaging at a lower electron accelerating voltage of 80 kV using the Sub-Angstrom Low-Voltage Electron Microscopy (SALVE) instrument (instrumental resolution is 0.78 Å) equipped with a chromatic (C_c_) and spherical (C_s_) aberration corrector. The C_c_-correction converts the background noise induced by inelastic scattering into imaging signals, improving the *S*/*N* ratio and dose efficiency.^36,37^ However, sample stability is also a problem at these lower accelerating voltages. Since inelastic interaction is increased, radiolysis effects will increase at 80 kV compared to 300 kV. Figure 3B shows, at an accelerating voltage of 80 kV, knock-on damage can be largely avoided. Thus, high-resolution imaging of Cu_3_(BHT) at 80 kV is made possible by balancing both damaging processes.

**Figure 4:**
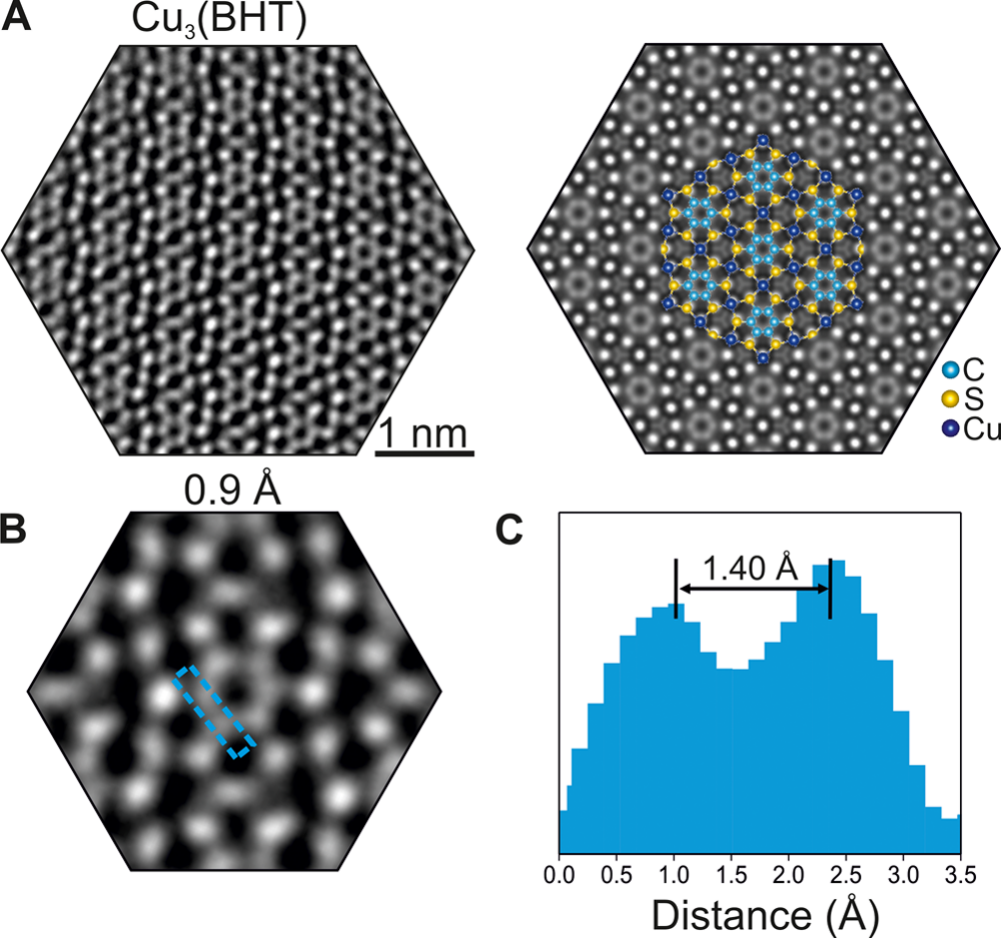
80 kV C_c_/C_s_-corrected HRTEM. (A) Experimental and simulated HRTEM images of Cu_3_(BHT), showing a qualitative fit. Acquisition dose: 3.2 × 10^3^ e^–^ Å^–2^. (B) Enlarged image of part A. (C) Intensity profile from the line-scan region in part B; the peak represents the center of the carbon atom, and the distance of the two peaks is 1.4 Å. Adapted with permission under a Creative Commons CC BY 4.0 from ref (18). Copyright 2023 Baokun Liang.

Due to the low scattering cross-section of carbon, unraveling the benzene rings in organic materials has been a long-standing challenge, thus, rarely reported.^6^ The C_c_/C_s_ correction combined with a reduced incident electron energy substantially increased the contrast in the HRTEM image, particularly at carbon sites (Figure 4A). Strikingly, we achieved an unprecedented resolution of 0.95 Å, enabling a clear distinction of neighboring carbon atoms (see Figure 4B and 4C, also Figure S7 in Supporting Information).

## Summary and Outlook

We have shown that systematically tailored structural changes of 2D c-MOFs strongly affected their physical properties and that these have a strong impact on their electron stability in high-resolution TEM experiments. The studied variations in the structure of the selected 2D c-MOFs thus provided further detailed insights into the factors that enhance the stability of 2D c-MOFs in TEM. Removing the hydrogen atoms contributed significantly to stabilizing the whole structure, while the changed atomic density of the material only had a minor influence on the measured stability under the electron beam. A breakthrough in sample stability has been achieved by modifying the structure to achieve enhanced conductivity, which drastically suppressed radiolysis, a major damaging mechanism of 2D c-MOFs. *Ab initio* molecular dynamics simulations of the knock-on events gave insight into the complex damaging pathways in Cu_3_(BHT). Note that due to the low conductivity of the other 2D c-MOF samples, knock-on simulations cannot reliably predict the overall stability of these structures in which radiolysis damage dominates. If in the future the sample thickness can be reduced to a monolayer, a sandwich structure with graphene on top and bottom of the monolayer 2D c-MOF sample might allow radiolysis effects to be largely suppressed. In this case, the manifold of different ejection pathways enabled through the tailored structure design will gain enormous importance.

The complexity of the simulation already became obvious in the four 2D c-MOFs shown here. Replacing the sulfur with oxygen, namely, going from Cu_3_(BHT) to Cu_3_(HHB) structure, a group of intermediate fragmentation pathways opened for a knock-on event on oxygen (see Figure S12 in Supporting Information). Compared with Cu_3_(BHT), intermediate fragmentation pathways had a much higher contribution to the overall knock-on damage in the other three 2D c-MOFs. Cu_3_(HHB) suffered the highest damage from intermediate pathways, but the Cu_3_(HHB), Cu_3_(HIB)_2_ structures were also affected. The Cu-pathway is also strongly influenced by the structure, *e.g*. in Cu_3_(HIB)_2_, and no ejection was observed following the impact on Cu at relevant energies.

To conclude, by obtaining 300 kV C_s_-corrected HRTEM images of the 2D c-MOFs, the dependence of resolution on the structural stability has been demonstrated. With improved resolution, the kagome structure of Cu_3_(HHB) has been directly observed. The high stability of Cu_3_(BHT) allowed us to image the structure at 80 kV acceleration voltage in the C_c_/C_s_-corrected SALVE instrument. Sub-Å resolution of the structure was reached together with elevated contrast, which allowed resolution of all structural features.

## Data Availability

All data supporting the findings of this study are available within the paper and its Supporting Information. Additional data related to this paper may be requested from the corresponding authors.

## Supporting Information Available

Thickness determination of MOFs, TEM methods, 2D c-MOF synthesis information, additional images demonstrating critical dose determination, resolution determination and thickness/defocus determination via simulation, demonstration of ctf correction, table including chemical information, lattice parameters and conductivity, table showing additional information about calculated cross sections, table displaying bond lengths within the four MOFs, information on calculation process, details on ejection thresholds and cross sections. (PDF)
