# Supplementary material for: Understanding the Electron Beam Resilience of Two-Dimensional Conjugated Metal–Organic Frameworks
Source: Nano Lett. 2024 Mar 1;24(10):3014–20. doi: 10.1021/acs.nanolett.3c04125 (PMC10941249; doi:10.1021/acs.nanolett.3c04125)
Supplement: Supplementary file 1 — nl3c04125_si_001.pdf [file nl3c04125_si_001.pdf]

Supplementary Information

Title

Understanding the Electron Beam Resilience of Two-Dimensional Conjugated Metal-Organic Frameworks

David Mücke,[1,*] Isabel Cooley,[2] Baokun Liang,[1] Zhiyong Wang,[3,4] SangWook Park,[4] Renhao Dong,[4,5] Xinliang Feng,[3,4] Haoyuan Qi,[1,4] Elena Besley[2,*] and Ute Kaiser[1,*]

[1] Central Facility for Materials Science Electron Microscopy, Universität Ulm, 89081 Ulm, Germany.

[2] School of Chemistry, University of Nottingham, University Park, Nottingham NG7 2RD, United Kingdom.

[3]Max Planck Institute for Microstructure Physics, 06120 Halle (Saale), Germany.

[4] Faculty of Chemistry and Food Chemistry & Center for Advancing Electronics Dresden, Technische Universität Dresden, 01062 Dresden, Germany.

[5] Key Laboratory of Colloid and Interface Chemistry of the Ministry of Education, School of Chemistry and Chemical Engineering, Shandong University, 250100 Jinan, China.

Corresponding authors: David.Muecke@uni-ulm.de Elena.Besley@nottingham.ac.uk; Ute.Kaiser@uni-ulm.de

This PDF file includes:

Figs. S1 to S13
Tables S1 to S7
References (1 to 23)
Additional Information on: TEM methods
Synthesis of 2D c-MOFs
Computational methods

Methods

Transmission electron microscopy. TEM experiments were performed on an image-side spherical aberration-corrected FEI Titan 80-300 operated at 300 kV, and the image-side spherical and chromatic aberration-corrected 20-80kV Sub-Angstrom Low-Voltage Electron Microscopy (SALVE) instrument at 80 kV. The FEI Titan is equipped with a CEOS hexapole C_s corrector that corrects the geometrical axial aberrations up to the 3rd-order. Data acquisition was conducted on a Gatan UltraScan1000 CCD camera. The SALVE C_s/C_c corrector adopts a quadrupole-octupole design, which corrects the geometrical axial aberrations up to the 5th-order, off-axial aberrations up to the 3rd order, and chromatic aberration. Data acquisition was conducted on a Ceta CMOS camera.

Critical dose determination from reflection fading. The critical dose was determined by monitoring the fading of the $<10\overline{1}0>$ reflections in the power spectra of C_s-corrected HRTEM image series of the 4 c-MOF samples. First order reflections where selected, due to the higher signal-to-noise ratio compared to higher order reflections. Additionally, first order reflections fade slower then higher order reflections. With that larger data-sets are obtainable. Image series were acquired with a constant dose rate: 1.2×10^3 e^- Å^{-2} s^{-1} for $Cu_3(HIB)_2$, $Cu_3(HHB)_2$, and $Cu_3(HHB)$, The acquisition time for each frame was 0.2 s; 2.9×10^3 e^- Å^{-2} s^{-1} for $Cu_3(BHT)$, acquisition time is 1s. A high contrast feature was used as a marker to monitor the sample drift due to the charging effect. The image acquisition continued until the total amorphization of the sample region, judged by the disappearance of first-order reflections. The power spectra in the image series were stacked into a power spectrum series. Via a custom-written Matlab script[36] (see Fig. S2 in Supporting Information), we selected first-order reflections and integrated their intensity in each frame. The intensity profile was plotted as a function of accumulated electron dose. The critical dose for total amorphization was determined when the intensity of the first-order reflections dropped to e^{-1} of their initial value. The electron dose was determined by the camera read-out, calibrated with a Faraday cup.

300 kV C_s- and 80 kV C_c/C_s-corrected-HRTEM imaging. For $Cu_3(HIB)_2$, $Cu_3(HHB)_2$, and $Cu_3(HHB)$, low-dose 300 kV C_s-corrected-HRTEM imaging was applied (TITAN 80-300). To minimise the damage to the sample before the TEM measurement starts in the region of interest, it is necessary to navigate on the TEM sample grid with a dose rate as low as possible, whilst ensuring sufficient contrast. To achieve this, the TEM was operated with an electron dose rate of 0.2 e^-/Å^{2}s. In search mode, an objective aperture was inserted to enhance the image contrast. To minimize the influence of other experimental parameters, the TEM was operated with the same beam diameter, dose rate, and apertures. Additionally, the pre-specimen shutter was used to minimize sample illumination. Once an area of interest was found, the beam was blanked. In the blanked state, the relevant settings for AC-HRTEM imaging were applied and the TEM stage was moved by the stage

controls so that the beam is no longer on the region of interest, but still remains close. The fine alignment of the beam, *e. g.* focus, was done before moving the blanked beam back onto the region of interest. Using this procedure, optimal imaging conditions were ensured whilst keeping the pre-illumination of the sample as small as possible.

The electron dose and pixel size for image acquisition of $Cu_3(HIB)_2$, $Cu_3(HHB)_2$, and $Cu_3(HHB)_2$, was 100 $e^- Å^{-2}$ (1.15 Å), 200 $e^- Å^{-2}$ (0.95 Å), and 200 $e^- Å^{-2}$ (0.74 Å), respectively. The 300 kV C_s-corrected-HRTEM and 80 kV C_c/C_s-corrected SALVE image of $Cu_3(BHT)$ share the same procedure. The acquisition dose for $Cu_3(BHT)$ is 5.6×10^3 $e^- Å^{-2}$ (at 300 kV, pixel size: 0.17 Å) and 3.2×10^3 $e^- Å^{-2}$ (at 80 kV, pixel size: 0.15 Å). Real-space averaging (18 unit cells) was applied to enhance the signal-to-noise ratio in experimental images, with a procedure described in.[37]**Image simulation.** HRTEM image simulations were performed with the abTEM package using the DFT relaxed 2D c-MOF structures.

Computational methods. *Ab initio* molecular dynamics (AIMD) was used to simulate transfer of energy following electron impact to the $Cu_3(BHT)$ MOF. A representative finite flake was used in place of the periodic structure to allow for the high-cost calculations. Each atom, in turn, was assigned the role of the primary knock-on atom (PKA), and in a simulation, the PKA was assigned a velocity equivalent to a selected transferred energy, which was approximated to be anisotropic and transferred perpendicular to the flake. Following time evolution simulations for a range of different transferred energies for each PKA, it was possible to extract ejection thresholds, E_d, for all relevant atoms. Atomic ejection cross-sections and fragment ejection cross-sections were calculated from E_d using the McKinley-Feshbach approach described in[15] and combined into total cross-sections with a weighted sum over all atoms of the relevant type, as in,[38] the inverse of which is the reported computational critical dose D_a. Further details are given in the Supporting Information.

Details on 2D c-MOF synthesis

$Cu_3(HIB)_2$: 2.04 mg (11.25 μmol) of copper acetate, 0.64 mg (6 μmol) of sodium carbonate, 0.75mg (2.6 μmol) of SDS were added into the 3 ml of deoxygenated, deionized water in 20 ml vial under nitrogen atmosphere. Subsequently, 2.09 mg (7.5 μmol) of HAB was added into a vial, and the reaction mixture in the vial was sealed under a nitrogen atmosphere. The sealed vial was then sonicated for 10 min to form bluish-gray colored dispersion. The dispersive mixture was treated at 50 °C under air to trigger the oxidation reaction at the air-water interface. After 12 hours, a free-standing HIB-Cu film with shiny reflection was observed on the water surface. The thickness of the film can be controlled by the concentration of copper acetate (5.625 to 16.875 μmol) and HAB (3.75 to 11.25 μmol). The synthesized film was transferred onto the Si/SiO2 substrate, TEM grid, or quartz substrate via the vertical deposition method. Film on solid substrate was gently rinsed with water and ethanol, thus dried under nitrogen atmosphere at 80 °C for 1 day.[1]

Synthesis of Cu_3(HHB): A 500 mL conical flask was used as the reaction container. $Cu(OAc)_2$ (480 mg, 2.61 mmol) and SDS (150 mg, 0.51 mmol) in 300 mL of water were prepared, followed by the addition of THQ (300 mg, 1.74 mmol) powder to the mixture. The mixture was sonicated for 30 minutes at 50 °C, then the reaction system was allowed to stay undisturbed for 10 h at 80 °C. The precipitate was collected and washed with water, ethanol, and acetone, and dried under vacuum for 12 h at 200 °C (75.5 % isolated yield). The reaction is scalable by equivalently increasing monomers and SDS in the mixture. [2]

Synthesis of $Cu_3(HHB)_2$: A 500 mL conical flask was used as the reaction container. $Cu(OAc)_2$ (480 mg, 2.61 mmol) and SDS (150 mg, 0.51 mmol) in 150 mL of water were prepared. Then, a solution of NaOH (150 mg, 3.75 mmol) in 150 mL of water was added to the as-prepared $Cu(OAc)_2$ and SDS solution, followed by the addition of THQ (300 mg, 1.74 mmol) powder to the mixture. The mixture was sonicated for 30 minutes at 50 °C, then the reaction system was allowed to stay undisturbed for 10 h at 25 °C. The precipitate was collected and washed with water, ethanol, and acetone, and dried under vacuum for 12 h at 100 °C (88.5 % isolated yield). The reaction is scalable by equivalently increasing monomers, SDS, and base in the mixture.

Synthesis of Cu_3(BHT): The thin film was prepared via a reaction between Cu(II) nitrate and BHT at the interface of dichloromethane–water. Under argon atmosphere, BHT was first dissolved in degassed dichloromethane to afford a saturated solution (0.24 mM). The solution (20 ml) of BHT was added to a sealed bottle filled with argon gas and then covered with degassed water (20 ml) to form an oil–water interface. The aqueous mixture of $Cu(NO_3)_2$ (5 mM) and NaBr (1 mM) was then added into the water gently and slowly (0.5 ml min^{-1}) by using a syringe pump. Formation of the film can be observed with naked eyes at the dichloromethane–water interface.[3]

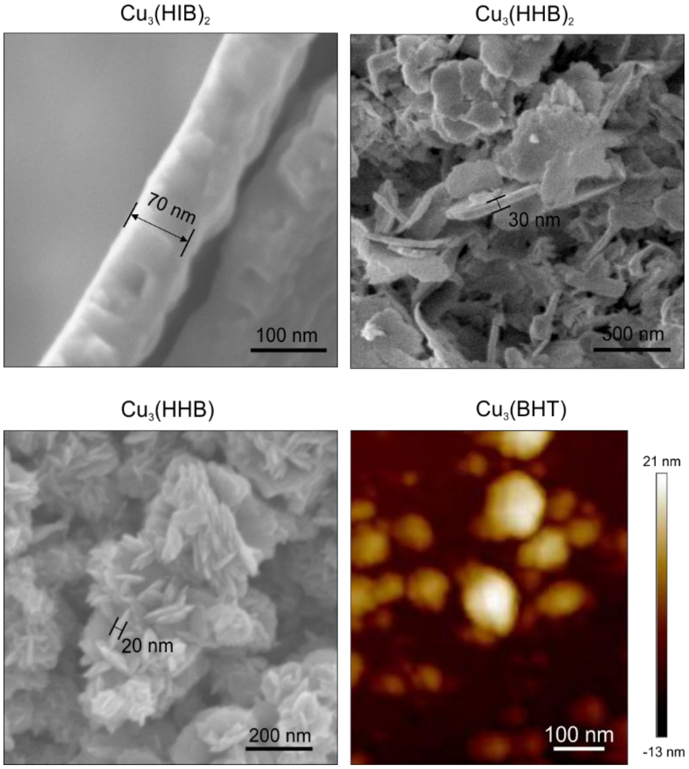

Fig S1. SEM and AFM images of 2D c-MOFs. The thickness of $Cu_3(HIB)_2$, $Cu_3(HHB)_2$, and $Cu_3(HHB)$ are determined from SEM image, the thickness values are labelled in the images. The thickness of $Cu_3(BHT)$ is measured by AFM, thickness range of the flakes are demonstrated by the scale bar. Reprinted with permission under a Creative Commons CC BY 4.0 from[23]. Copyright 2023 Baokun Liang.

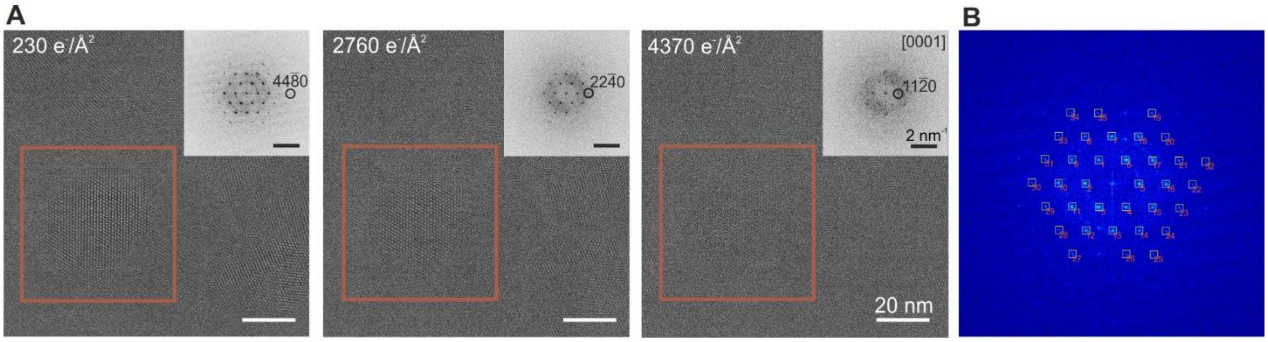

Fig S2. Dose series and critical dose analysis. (A) Snapshots from the dose series of $Cu_3(HIB)_2$ after exposing to an increasing amount of electron dose. The electron dose rate was constant: 1150 $e^- Å^{-2} s^{-1}$. Insets: FFT patterns from the boxed region. The circles mark the highest order reflections remaining in the FFT. Scale bar: 2 nm^{-1}. (B) the Bragg reflections positions determined by selecting the spot position on the first FFT of the image series in the homemade Matlab script, each spot is encircled by a box, the same positions are applied for the whole series. To obtain the critical dose, the intensity of the $<10\bar{1}0>$ reflections is measured in the whole image stack.

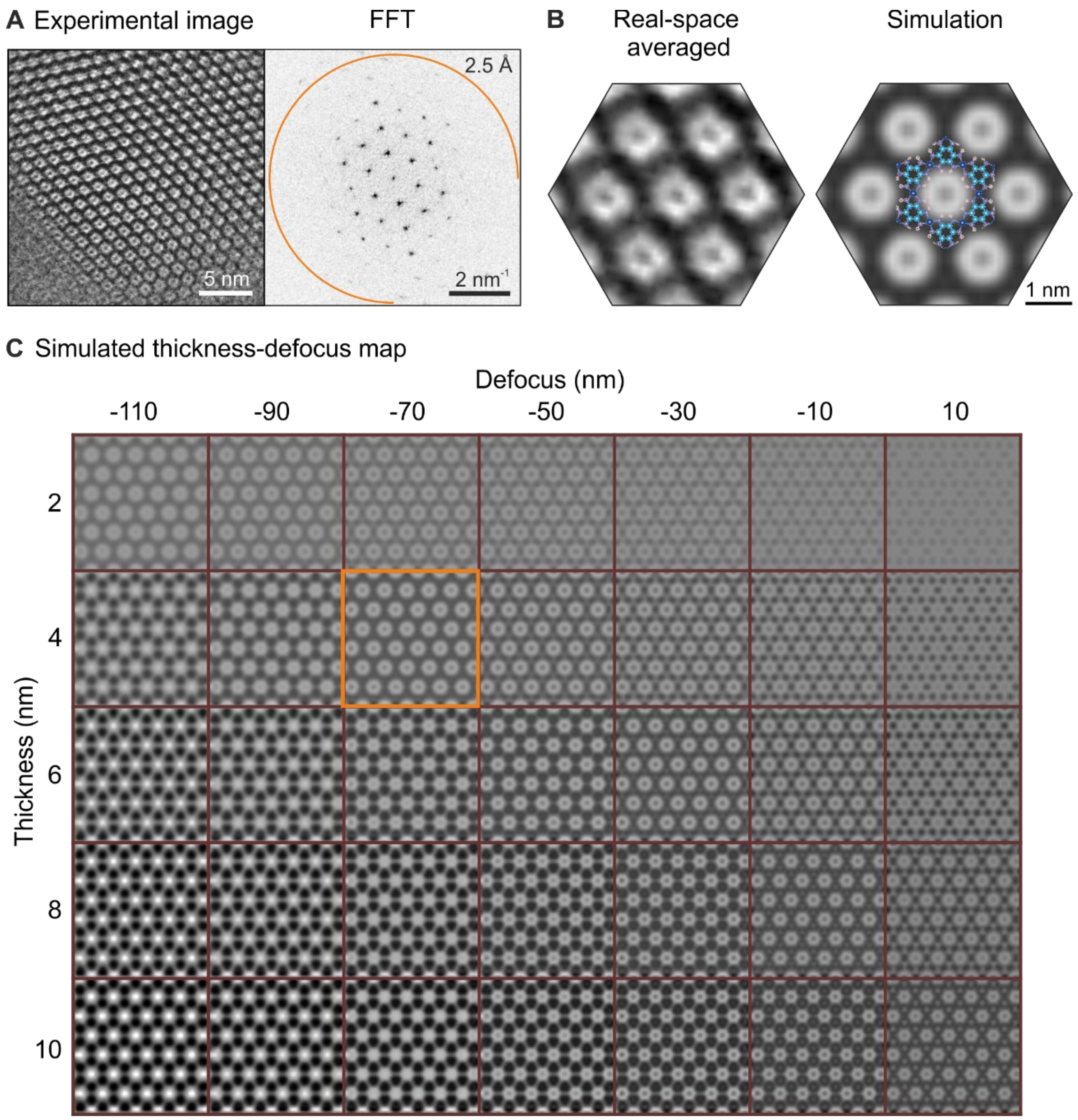

Fig S3. Cs-corrected HRTEM imaging of $Cu_3(HIB)_2$ and image simulation. (A) Experimental image in [0001] direction, acquired at 300 kV with a dose of 100 $e^- Å^{-2}$. The circle in the FFT pattern denotes the highest information transfer. (B) Comparison between experimental and simulated images. Real-space averaging has been conducted to enhance the signal-to-noise ratio. (C) Simulated thickness-defocus map. The best fitting simulated image is highlighted (thickness: 4 nm, defocus: -70 nm).

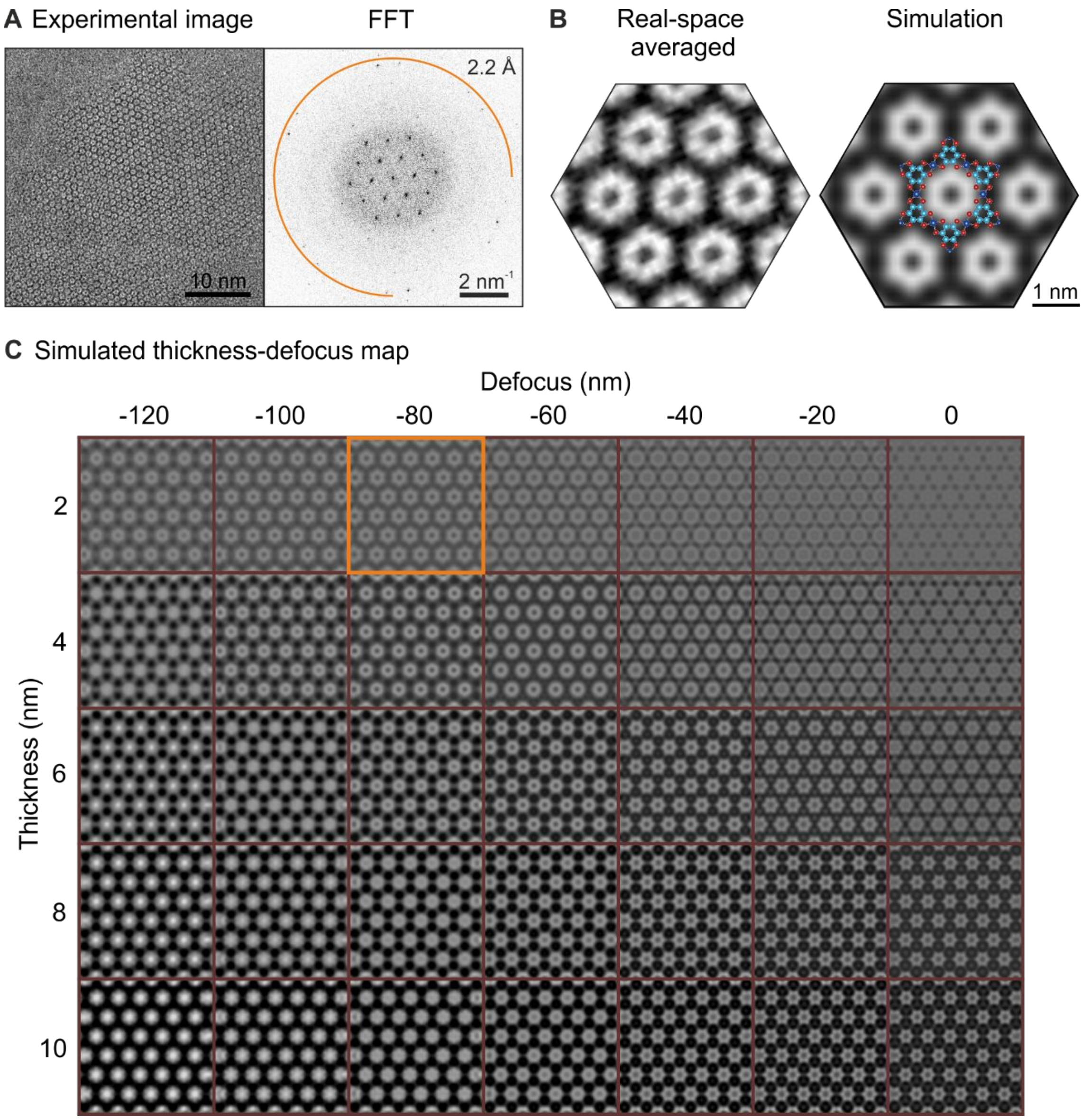

Fig S4. Cs-corrected HRTEM imaging of $Cu_3(HHB)_2$ and image simulation. (A) Experimental image in [0001] direction, acquired at 300 kV with a dose of 200 e^- $Å^{-2}$. The circle in the FFT pattern denotes the highest information transfer. (B) Comparison between experimental and simulated images. Real-space averaging has been conducted to enhance the signal-to-noise ratio. (C) Simulated thickness-defocus map. The best fitting simulated image is highlighted (thickness: 2 nm, defocus: -80 nm).

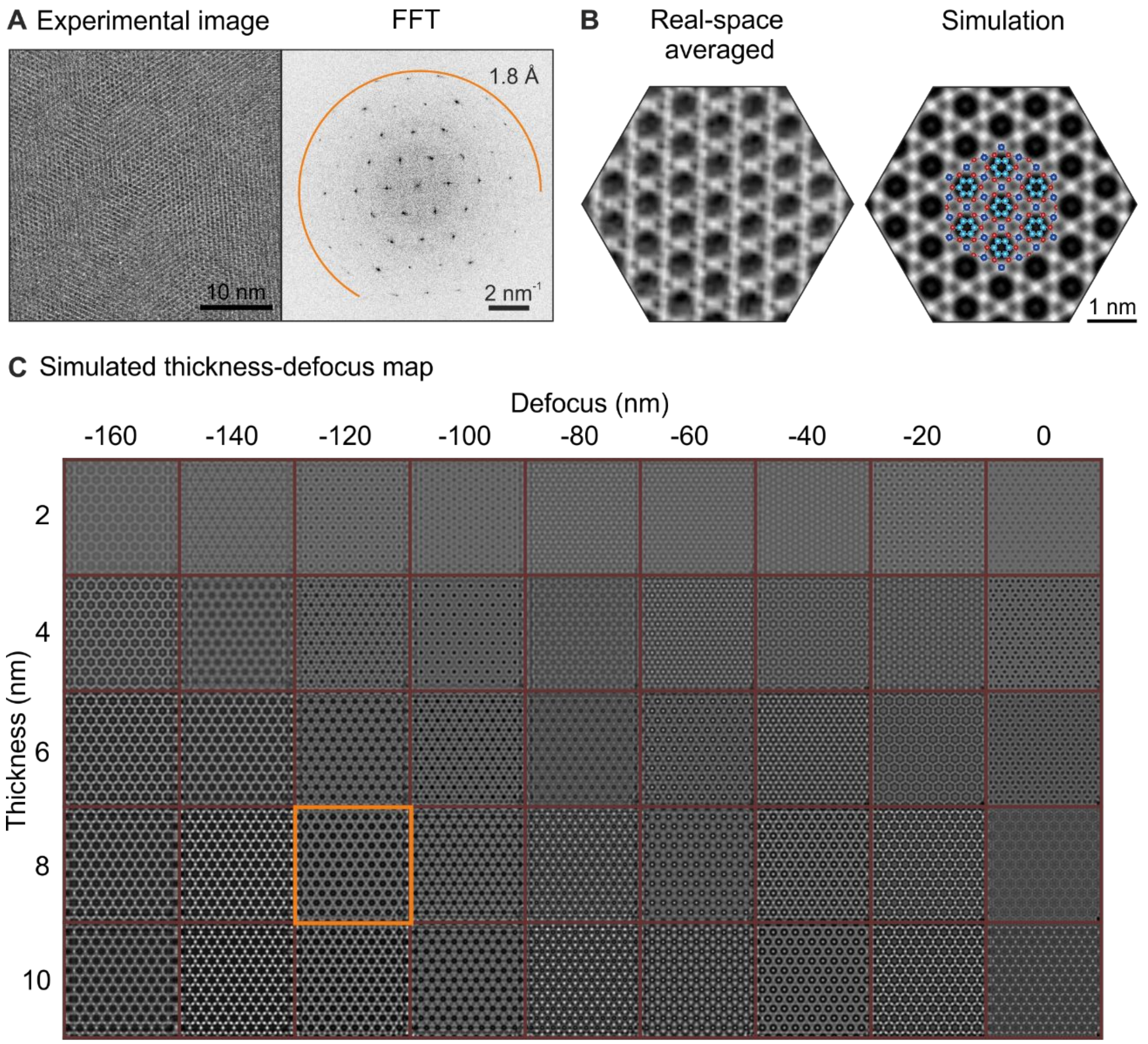

Fig S5. Cs-corrected HRTEM imaging of Cu_3(HHB) and image simulation. (A) Experimental image in [0001] direction, acquired at 300 kV with a dose of 200 e^{-} $Å^{-2}$. The circle in the FFT pattern denotes the highest information transfer. (B) Comparison between experimental and simulated images. Real-space averaging has been conducted to enhance the signal-to-noise ratio. (C) Simulated thickness-defocus map. The best fitting simulated image is highlighted (thickness: 8 nm, defocus: -120 nm).

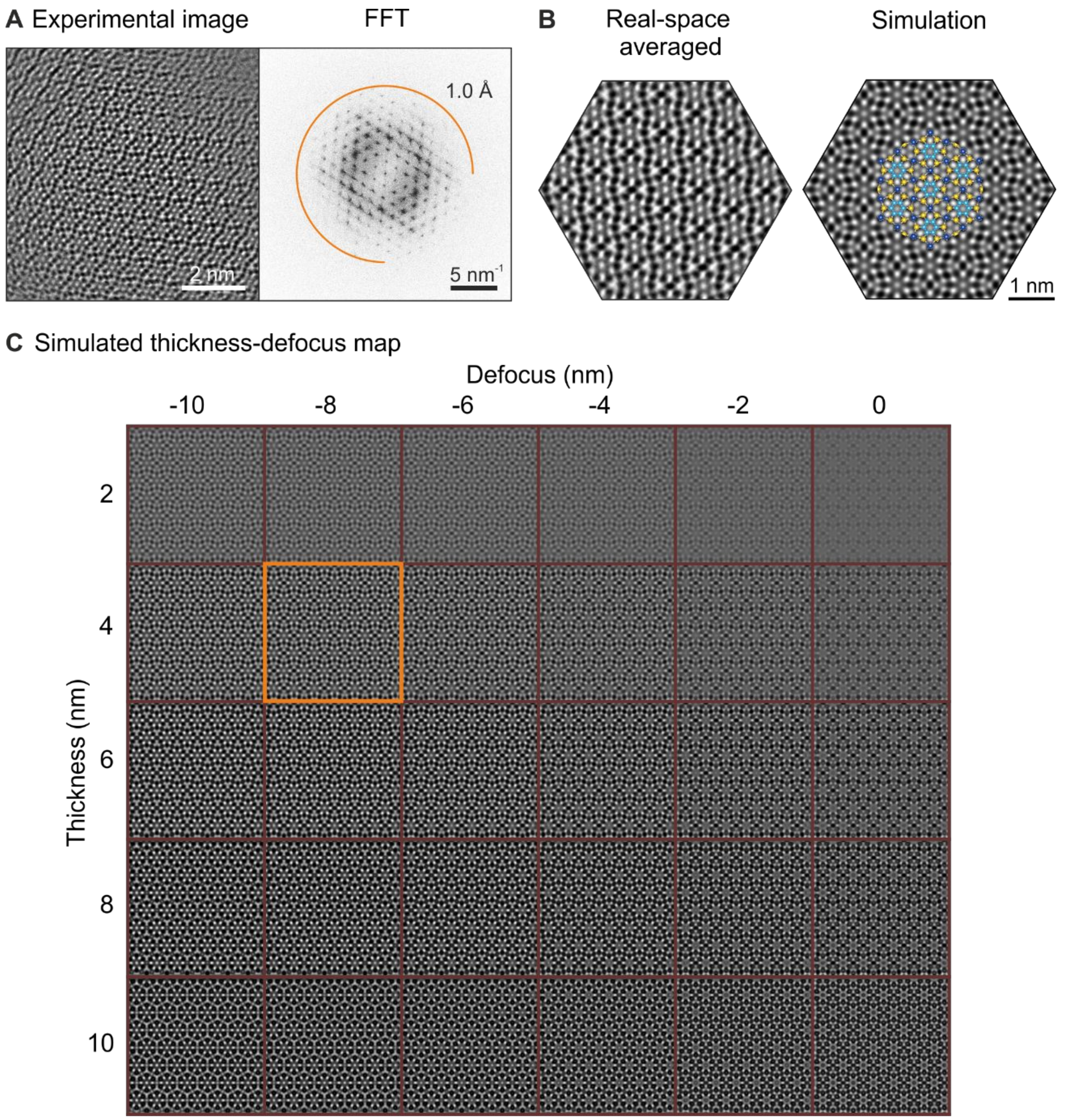

Fig S6. Cs-corrected HRTEM imaging of Cu_3(BHT) and image simulation. (A) Experimental image in [0001] direction, acquired at 300 kV with a dose of 5.6 × 10^3 e^- $Å^{-2}$. The circle in the FFT pattern denotes the highest information transfer. (B) Comparison between experimental and simulated images. Real-space averaging has been conducted to enhance the signal-to-noise ratio. (C) Simulated thickness-defocus map. The best fitting simulated image is highlighted (thickness: 4 nm, defocus: -8 nm).

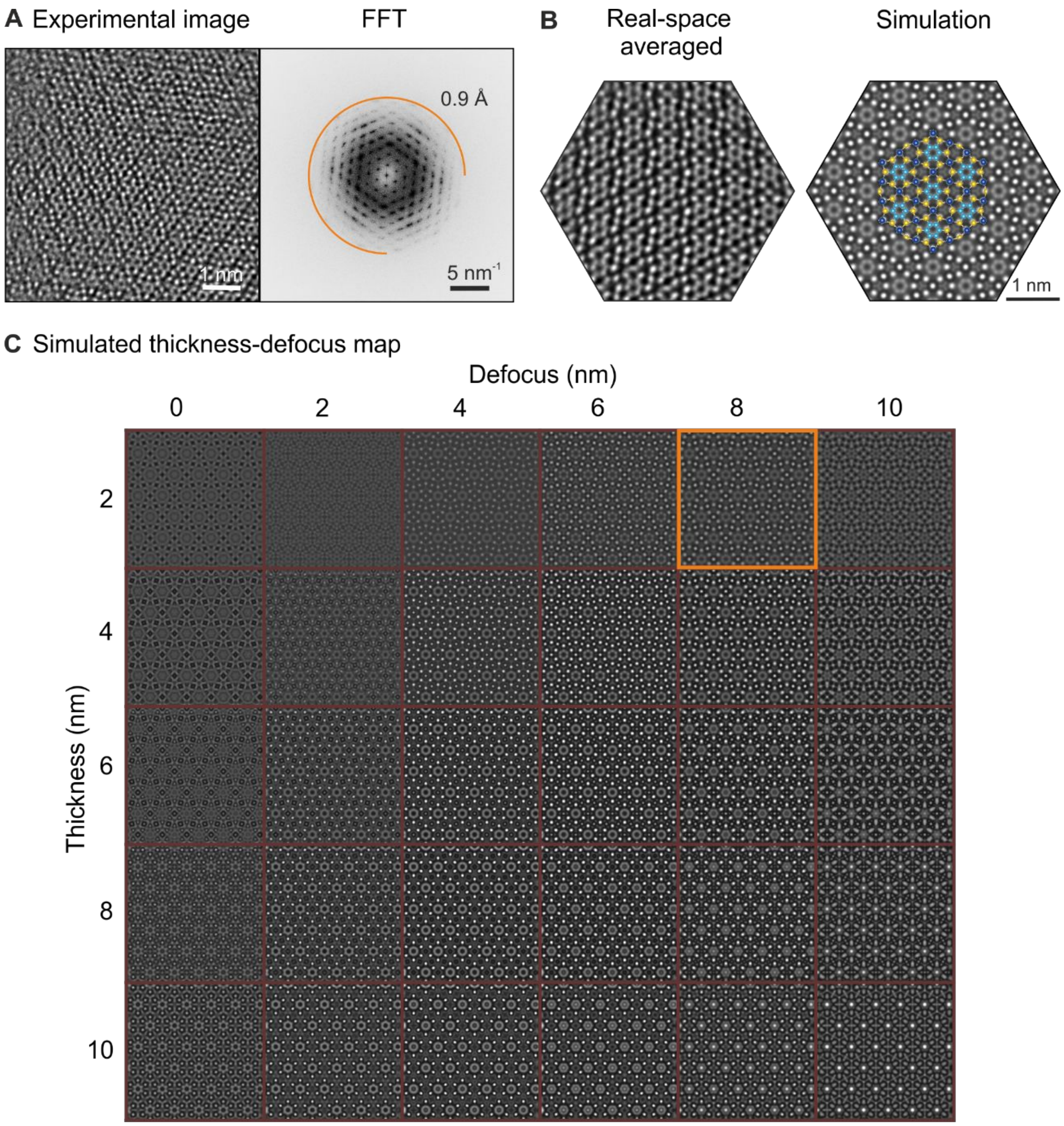

Fig S7. Cs+Cc-corrected HRTEM imaging of Cu_3(BHT) and image simulation. (A) Experimental image in [0001] direction, acquired at 300 kV with a dose of 3.2×10^3 e^- $Å^{-2}$. The circle in the FFT pattern denotes the highest information transfer. (B) Comparison between experimental and simulated images. Real-space averaging has been conducted to enhance the signal-to-noise ratio. (C) Simulated thickness-defocus map. The best fitting simulated image is highlighted (thickness: 2 nm, defocus: 8 nm).

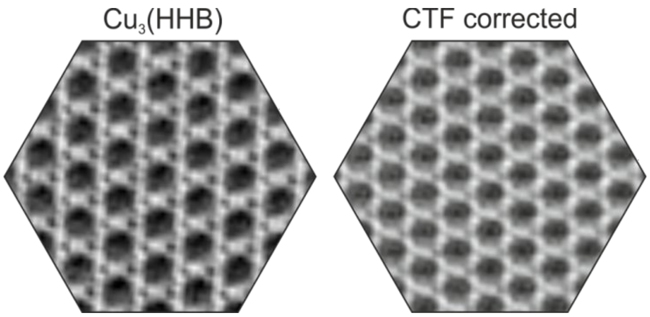

Fig S8. Cs-corrected HRTEM imaging of Cu_3(HHB) and contrast transfer function (CTF) corrected image. On the left, it is the HRTEM image of Cu_3(HHB), according to the simulation in Fig S5, the image condition is at defocus: -120 nm. On the right is the CTF corrected image processed by QFocus [4]. Images in [0001] direction.

Table S1. Ligand, symmetry, lattice parameters, and conductivities of the 2D c-MOFs.

HAB: hexaaminobenzene; HIB: hexaiminobenzene; HHB: hexahydroxybenzene; BHT: benzenehexathiol.

Ligand	2D MOFs	Chemical formula	Symmetry	Lattice parameters	Electrical conductivity at 298 K	Ref
HAB	$Cu_3(HIB)_2$	$Cu_3(C_6N_6H_6)_2$	honeycomb	*a*=13.5 Å<br>*c*=3.3 Å	13 S cm^{-1}	5
HHB	$Cu_3(HHB)_2$	$Cu_3(C_6O_6)_2$	honeycomb	*a*=13.1 Å<br>*c*=3.0 Å	7.3 x 10^{-8} S cm^{-1}	6
HHB	Cu_3(HHB)	$Cu_3(C_6O_6)$	hexagonal	*a*=7.5 Å<br>*c*=2.9 Å	2.6 ×10^{-2} S cm^{-1}	Unpublished data
BHT	Cu_3(BHT)	$Cu_3(C_6S_6)$	hexagonal	*a*=8.7 Å<br>*c*=3.5 Å	2500 S cm^{-1}	7

Table S2. Cross-sections at 300 keV for each atom in $Cu_3(HIB)_2$ and $Cu_3(HHB)_2$. Also combined cross-section per Cu atom for the whole MOFs, experimental electron doses and calculated electron doses per Cu atom.

**$Cu_3(HIB)_2$**				**$Cu_3(HHB)_2$**			
Impacted atom	Atoms per Cu	Ejected group	σ_d /barn	Impacted atom	Atoms per Cu	Ejected group	σ_d /barn
Cu	1	Cu	28.08	Cu	1	Cu	10.45
N	4	NH	45.56	O	4	O	33.54
C	4	CNH	22.13	C	4	CO	35.54
H	4	H	42.97				
Total σ_d per Cu /barn		470.75		Total σ_d per Cu /barn		286.77	
Experimental electron dose /ke$^-$ Å^{-2}		4.5 ± 0.4		Experimental electron dose /ke$^-$ Å^{-2}		8.3 ± 0.2	
Calculated dose per Cu /ke$^-$ Å^{-2}		212.43		Calculated dose per Cu /ke$^-$ Å^{-2}		348.71	

Table S3. Bond lengths in the 2D c-MOFs. Reprinted with permission under a Creative Commons CC BY 4.0 from[23]. Copyright 2023 Baokun Liang.

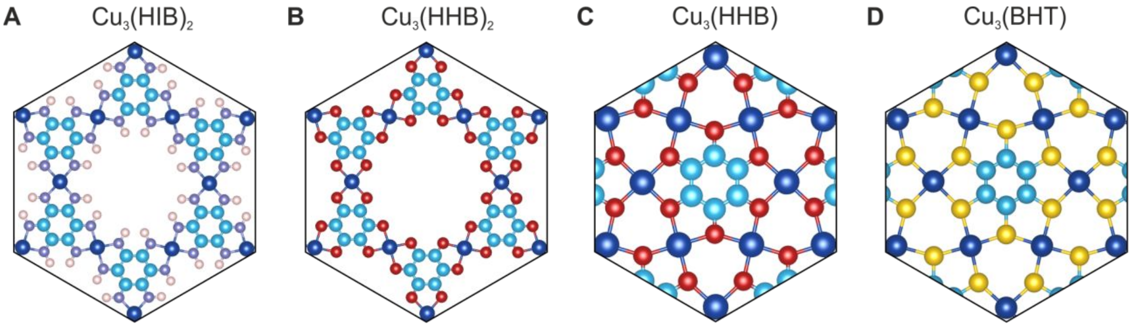

A

Bond	Distance (Å)
C-C	1.4
C-N	1.3
Cu-N	2.0
N-H	1.1

B

Bond	Distance (Å)
C-C	1.4
C-O	1.3
Cu-O	2.0

C

Bond	Distance (Å)
C-C	1.4
C-O	1.4
Cu-O	2.0

D

Bond	Distance (Å)
C-C	1.4
C-S	1.8
Cu-S	2.4

Further Details on Computational Methods

For the computational part of this study, we calculate cross sections, σ_d, for permanent knock-on ejection of atoms or fragments from the four 2D c-MOFs. From these we determine computational critical electron doses for amorphization at 300 keV. Considering the time between electron impacts in TEM, it is supposed that damage short of permanent ejection caused by an impact is repaired before any subsequent impact, achieving restoration of equilibrium, and thus crystallinity,[8,9] whereas permanent ejection is treated as a precursor to full amorphization. Only direct knock-on damage is considered computationally: the methods make no consideration of electron dynamics, neglecting damage caused by inelastic scattering events. The impact of inelastic compared to knock-on damage has previously been shown to decrease as beam energy increases, faster electrons having less time to induce excitation.[10,11] Knock-on damage is expected to be the dominant pathway at a beam energy of 300 keV, and more generally for conductive materials, in which excited electrons relax easily to the ground state between electron impacts.[8,10,11]

Spin polarised ab initio molecular dynamics (AIMD) at the B3LYP/6-31G* level of theory was employed using the ORCA software package[12–14] to simulate the effects of energy transfer following electron impacts on the constituent atoms of the four MOFs. A method's performance varies depending on the system and chemical property to which it is applied; B3LYP/6-31G* is widely used, with good general applicability.[15] Indeed, applied to calculation of a range of chemical properties of organic systems, it is not atypical for 6-31G-type basis sets to equal or outperform more computationally demanding competitors, neither is it uncommon for only a small effect to be observed following expansion of the basis set by addition of diffuse functions (6-31+G* or 6-31++G*).[15] When it comes to the particular challenge of transition metal modelling, the errors associated with DFT methods are notably higher than for organic molecules.[16] Hybrid functionals such as B3LYP, while performing better than their non-hybrid counterparts for main group systems,[17,18] have come under criticism for their ability to model transition metal dimers[17] and, to a lesser extent, small metal-ligand complexes.[18] Among the transition metals, copper suffers from this issue comparatively little,[17,18] although 6-31G-type basis sets have met some criticism specific to copper, regarding their ability to calculate the bond length of the copper dimer.[19] None of the four MOFs contains a Cu-Cu bond, and such bonds are observed rarely if at all over the course of the dynamics simulations. In previous comparison of a range of methods and basis sets for modelling transition metal-ligand complexes,[16,20] B3LYP/6-31G** showed reasonable performance including for copper complexes. The 6-31G** basis set differs from 6-31G* only in the inclusion of polarisation functions on hydrogen atoms. Although by no means the most accurate computational method for copper-organic complexes,[20] B3LYP/6-31G* is used as an appropriate compromise between accuracy for the organic part of the system, accuracy for the metal, and of course the substantial cost of AIMD.

Even employing a relatively modest level of theory, AIMD with large systems is prohibitively expensive: representative finite flakes were used in place of periodic structures, reducing cost to a computationally manageable level. The selected flakes are shown in Figure S9, with dangling bonds capped with hydrogen atoms and. For convenience in discussion of the identify of impacted atoms and ejected fragments, atoms which are active in the simulations, that is, atoms which at any point form part of an ejected fragment are labelled numerically, with impacted atoms labelled in red. Within the flakes, each atom type in turn acted as the primary knock-on atom (PKA), to which energy was transferred. All atoms of any given element in the MOFs are symmetrically equivalent in the periodic structure, so only one atom of each element was simulated in each flake. In cases where flake atoms are not all symmetrically equivalent, atoms whose environment best approximated bulk were selected. Based on initial tests, selected edge atoms of each flake

were immobilised during simulations to prevent excessive translation of the flakes. In Figure S9 atoms circled in blue and any hydrogen atoms connected to them, are the immobilised atoms.

Prior to a dynamics simulation, initial velocities were assigned to the flake atoms: a velocity perpendicular to the plane of the flake equivalent to a selected transferred energy for the PKA, and zero velocity for all other atoms and directions. The system was allowed to propagate using molecular dynamics with timesteps of 0.5 fs until it was judged that any ejection or lack thereof was permanent. This was repeated for a range of energies, with ejection of an atom or fragment, or non-ejection, observed in each case. The range was gradually narrowed until ejection thresholds, E_d, had been obtained to specified precision. Taking the energies used as T_{max} the maximum possible energy transferred to a static atom for a given beam energy as calculated by equation 1,[21] the precision was 0.1 keV beam energy.

$$T = \frac{2ME(E + 2m_e c^2)}{(M + m_e)^2 c^2 + 2ME} sin^2(\theta/2) \qquad (1)$$

In equation 1, M is the mass of the atom, E is the electron kinetic energy, m_e is the mass of an electron, c is the speed of light and θ is the electron scattering angle. T_{max} occurs when $\theta = 180°$ and $sin^2(\theta/2) = 1$.

Using the calculated E_d for ejections observed following impacts on all flake atoms (with the exception of the capping H atoms which do not exist in the real structure), atomic and fragment ejection cross-sections were calculated as described by Skowron et al,[9] using the McKinley-Feshbach formula,[22] which yields equation 2, where e is electron charge and β is the ratio of electron velocity to the speed of light. The method[9] includes consideration of the initial velocity distribution of lattice atoms using the Maxwell-Boltzmann distribution.

$$\sigma_d = 4\pi\left(\frac{ze^2}{4\pi\varepsilon_0 2m_e c^2}\right)^2 \frac{1-\beta^2}{\beta^4}\left[\frac{T_{max}}{E_d} - 1 - \beta^2 \ln\left(\frac{T_{max}}{E_d}\right) + \pi\frac{ze^2}{\hbar c}\beta\left(2\left(\frac{T_{max}}{E_d}\right)^{\frac{1}{2}} - \ln\frac{T_{max}}{E_d} - 2\right)\right] \qquad (2)$$

Individual ejection cross sections were combined into total ejection cross-sections for each of the four MOFs with a weighted sum over all atoms of the relevant type, as in Chamberlain et al,[21] where total cross-sections for damage are given per molecule. For the periodic structures, total cross sections were here calculated per copper atom, which is equivalent to per empirical formula unit. Critical dose for amorphization per copper atom, the inverse of the total cross section per copper atom, was then obtained.

Supplementary Computational Data

In this section is given a breakdown of individual ejection events, and computational thresholds and cross sections not given in the main text. Figures S10, S11 and S12 detail observed fragmentation pathways following impacts on the constituent atoms of the $Cu_3(HIB)_2$, $Cu_3(HHB)_2$ and $Cu_3(BHT)$ finite flakes respectively, with Tables S4, S5, S6 and S7 contain numerical values of this information for all four MOFs. It is observed that impacts on Cu alead to only the primary ejection pathway (no intermediate fragmentation pathways) for all MOFs. The same is true of impacts on N and H in $Cu_2(HIB)$ and S in $Cu_2(BHT)$, but impacts on the remaining non-metals lead to intermediate fragmentation pathway contribution. In addition, figure S5 displays for $Cu_3(HIB)_2$ the plot of computational knock-on cross sections with a larger y axis maximum. This illustrates the very high hydrogen ejection threshold for this MOF.

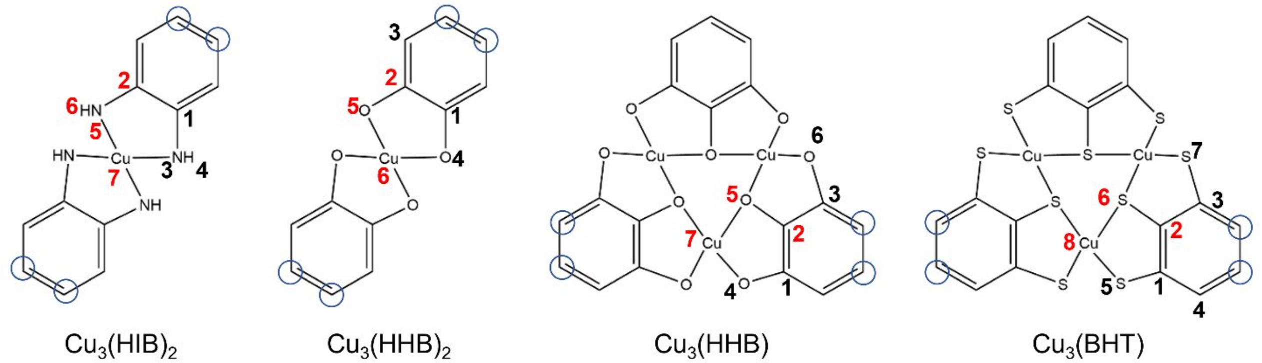

Fig S9. The representative fragments used in simulations for each of the four MOFs. Atoms which form parts of ejected fragments over the course of the simulations are labelled numerically for convenience. Atoms whose labels are in red are the impacted atoms. Atoms which are circled in blue, and hydrogen atoms connected to them, are frozen during the simulations.

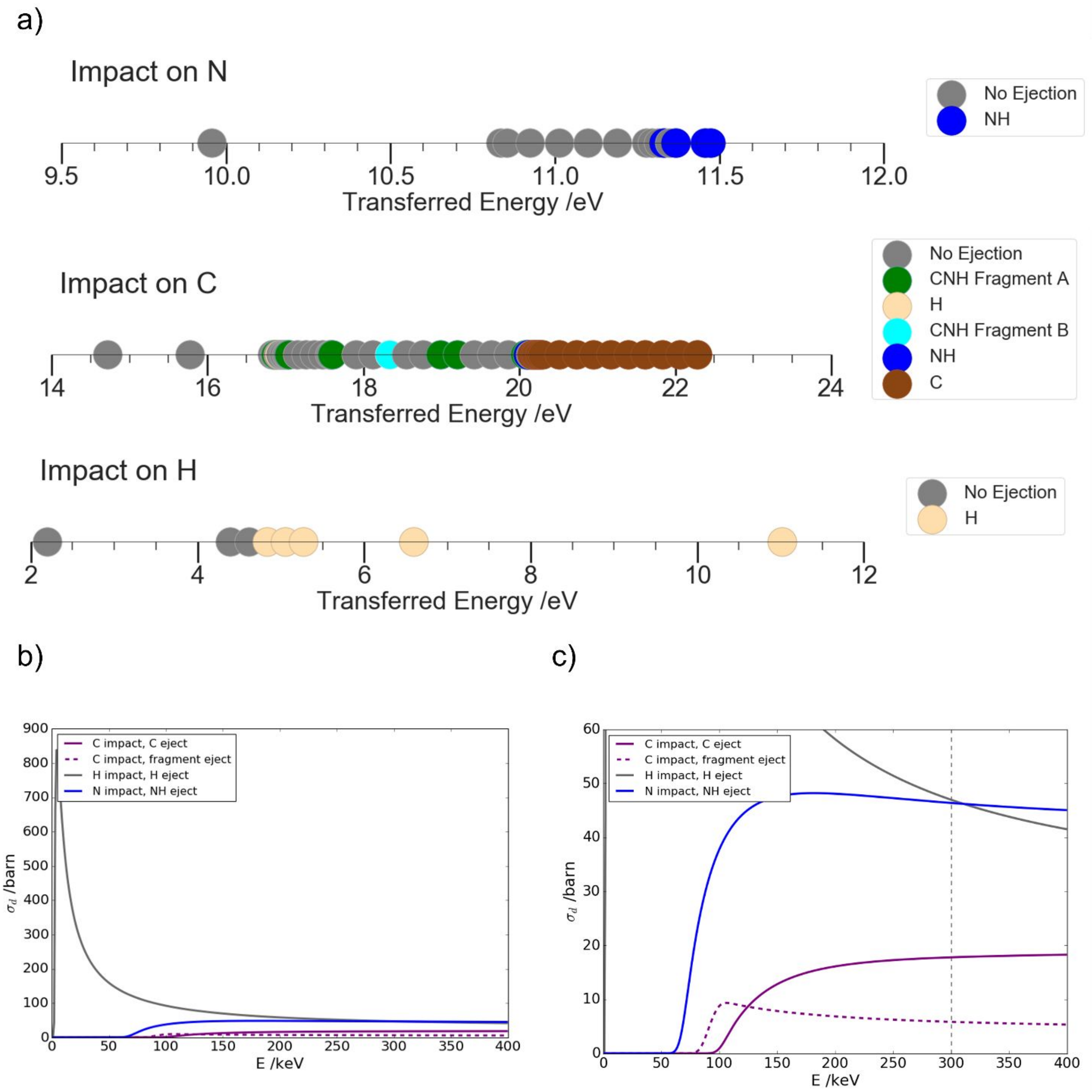

Fig S10. Details of $Cu_3(HIB)_2$ ejection thresholds and cross sections. a) Number lines showing fragmentation events over a range of transferred energies. b) and c) Plots of computational knock-on cross sections following impacts on each atom type, with different maximum y-values plotted. Using the number system established in Fig. S9, the ejected atoms and fragments are identified as follows. NH: N5H6; H: H6; C: C2; CNH fragment A: C2N5H6; CNH fragment B: C1N3H4.

a)

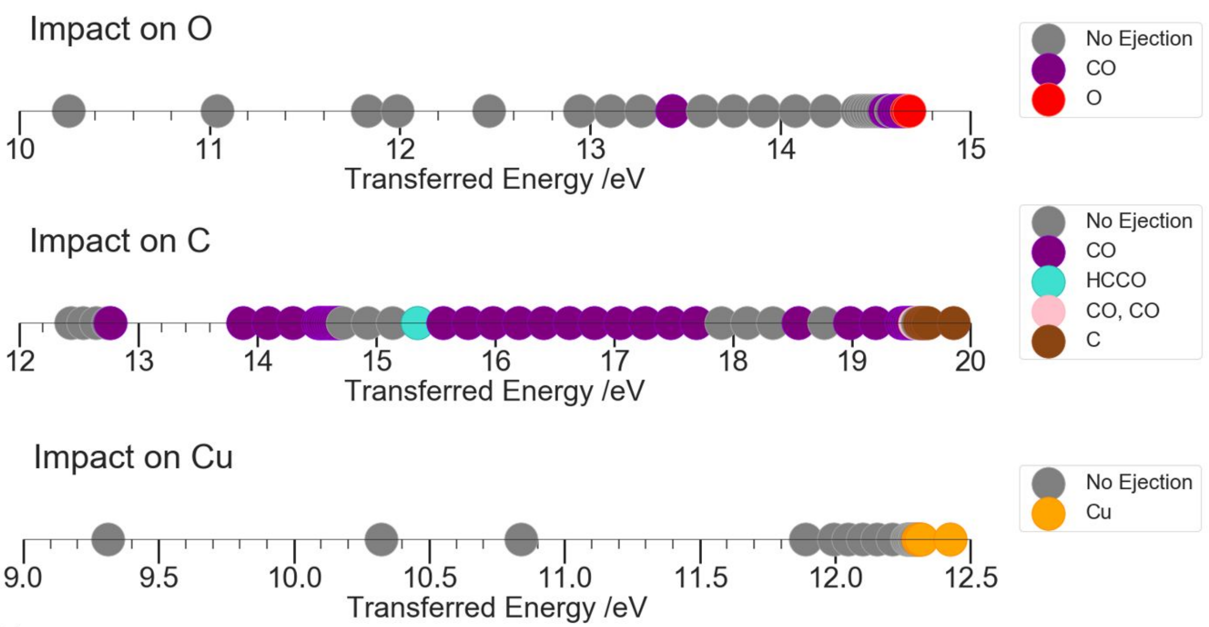

b)

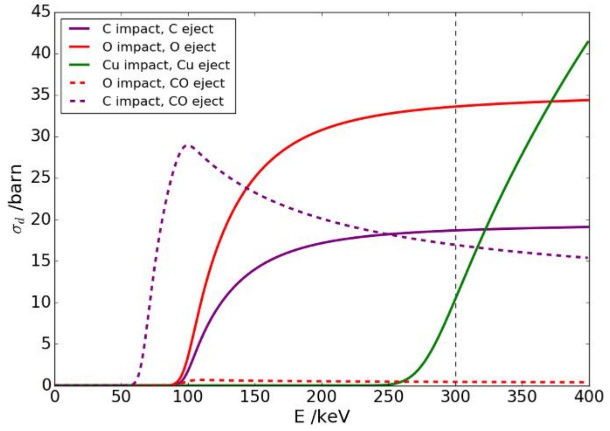

Fig S12. Details of $Cu_3(HHB)_2$ ejection thresholds and cross sections. a) Number lines showing fragmentation events over a range of transferred energies. b) Plots of computational knock-on cross sections following impacts on each atom type, with different maximum y-values plotted. Using the number system established in Fig. S9, the ejected atoms and fragments are identified as follows. O: O5; C: C2; CO: C2O5; HCCO: C2C3HO5; CO, CO: C2O5, C1O4.

a)

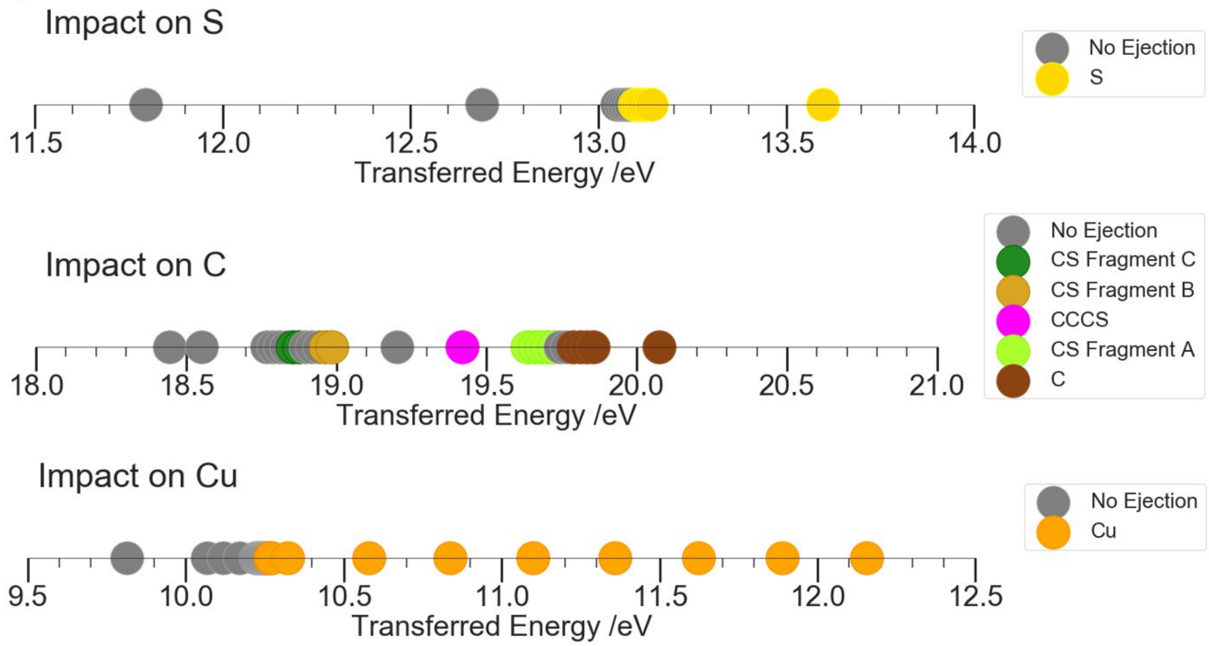

b)

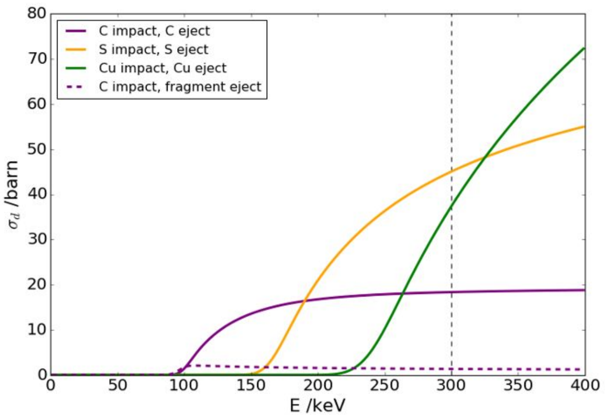

Fig S13. Details of Cu_3(BHT) ejection thresholds and cross sections. a) Number lines showing fragmentation events over a range of transferred energies. b) Plots of computational knock-on cross sections following impacts on each atom type, with different maximum y-values plotted. Using the number system established in Fig. S9, the ejected atoms and fragments are identified as follows. S: S6; C: C2; Cu: Cu8; CCCS: C2C4C1S5; CS fragment A: C1S5; CS fragment B: C2S5; CS fragment C: C3S7.

Table S4. Thresholds and cross sections for impacts on the atoms in the $Cu_3(HIB)_2$ flake. Using the system established in Fig. S9, the atoms and fragments directly referred to are as follows. N: N15; NH: N5H6; C: C2; H: H6.

Impacted atom	Ejected atom(s)	Transferred energy /eV	Approx. beam energy /keV	σ (300 keV) /barn	Total σ from impact
N	NH	11.33	67.8	46	46
C	Fragments	16.88	85.2	6	24
	C	20.16	100.4	18	
H	H	4.83	2.2	47	47
Cu	None			0	0

Table S5. Thresholds and cross sections for impacts on the atoms in the $Cu_3(HHB)_2$ flake. Using the system established in Fig. S9, the atoms and fragments directly referred to are as follows. O: O5; C: C2; CO: C2O5.

Impacted atom	Ejected atom(s)	Transferred energy /eV	Approx. beam energy /keV	σ (300 keV) /barn	Total σ from impact
O	CO	14.55	96.9	0.4	34
	O	14.66	97.6	34	
C	CO	12.76	65.6	17	36
	C	19.57	97.7	19	
Cu	Cu	12.31	277.8	10	10

Table S6. Thresholds and cross sections for impacts on the atoms in the Cu_3(HHB) flake. Using the system established in Fig. S9, the atoms and fragments directly referred to are as follows. O: O8; CO (A): C5O8; CO (B): C4O7; CO (C): C6O9; CO (D): C5O7; C: C5; Cu: Cu7.

Impacted atom	Ejected atom(s)	Transferred energy /eV	Approx. beam energy /keV	σ (300 keV) /barn	Total σ from impact
O	CO (A)	11.69	79.1	19	48
	O	16.18	106.8	29	
C	CO (C)	13.49	69.1	3	33
	CO (other)	14.53	74.1	9	
	C	18.64	93.4	20	
Cu	Cu	11.68	266	17	17

Table S7. Thresholds and cross sections for impacts on the atoms in the Cu_3(BHT) flake. Using the system established in Fig. S9, the atoms and fragments directly referred to are as follows. S: S6; C: C2; Cu: Cu8.

Impacted atom	Ejected atom(s)	Transferred energy /eV	Approx. beam energy /keV	σ (300 keV) /barn	Total σ from impact
S	S	13.10	164.5	45	45
C	Fragments	18.96	94.9	1	20
	C	19.79	98.7	18	
Cu	Cu	10.27	238.9	37	37

References

(1) Park, S.; Zhang, Z.; Qi, H.; Liang, B.; Mahmood, J.; Noh, H.-J.; Hambsch, M.; Wang, M.; Wang, M.; Ly, K. H.; Wang, Z.; Weidinger, I. M.; Zhou, S.; Baek, J.-B.; Kaiser, U.; Mannsfeld, S. C. B.; Feng, X.; Dong, R. In-Plane Oriented Two-Dimensional Conjugated Metal–Organic Framework Films for High-Performance Humidity Sensing. *ACS Mater. Lett.* **2022**, *4*, 1146–1153.

(2) Wang, Z.; Wang, G.; Qi, H.; Wang, M.; Wang, M.; Park, S.; Wang, H.; Yu, M.; Kaiser, U.; Fery, A.; Zhou, S.; Dong, R.; Feng, X. Ultrathin Two-Dimensional Conjugated Metal–Organic Framework Single-Crystalline Nanosheets Enabled by Surfactant-Assisted Synthesis. *Chem. Sci.* **2020**, *11*, 7665–7671.

(3) Huang, X.; Sheng, P.; Tu, Z.; Zhang, F.; Wang, J.; Geng, H.; Zou, Y.; Di, C.; Yi, Y.; Sun, Y.; Xu, W.; Zhu, D. A Two-Dimensional π–d Conjugated Coordination Polymer with Extremely High Electrical Conductivity and Ambipolar Transport Behaviour. *Nat. Commun.* **2015**, *6*, 7408.

(4) Wan, W.; Hovmöller, S.; Zou, X. Structure Projection Reconstruction from Through-Focus Series of High-Resolution Transmission Electron Microscopy Images. *Ultramicroscopy* **2012**, *115*, 50–60.

(5) Dou, J. H.; Sun, L.; Ge, Y.; Li, W.; Hendon, C. H.; Li, J.; Gul, S.; Yano, J.; Stach, E. A.; Dincă, M. Signature of Metallic Behavior in the Metal-Organic Frameworks M3(Hexaiminobenzene)2 (M = Ni, Cu). *J. Am. Chem. Soc.* **2017**, *139* , 13608–13611.

(6) Park, J.; Hinckley, A. C.; Huang, Z.; Feng, D.; Yakovenko, A. A.; Lee, M.; Chen, S.; Zou, X.; Bao, Z. Synthetic Routes for a 2D Semiconductive Copper Hexahydroxybenzene Metal-Organic Framework. *J. Am. Chem. Soc.* **2018**, *140*, 14533–14537.

(7) Huang, X.; Zhang, S.; Liu, L.; Yu, L.; Chen, G.; Xu, W.; Zhu, D. Superconductivity in a Copper(II)-Based Coordination Polymer with Perfect Kagome Structure. *Angew. Chemie - Int. Ed.* **2018**, *57*, 146–150.

(8) Santana, A.; Zobelli, A.; Kotakoski, J.; Chuvilin, A.; Bichoutskaia, E. Inclusion of Radiation Damage Dynamics in High-Resolution Transmission Electron Microscopy Image Simulations: The Example of Graphene. *Phys. Rev. B* **2013**, *87*, 94110.

(9) Skowron, S. T.; Lebedeva, I. V; Popov, A. M.; Bichoutskaia, E. Approaches to Modelling Irradiation-Induced Processes in Transmission Electron Microscopy. *Nanoscale* **2013**, *5*, 6677–6692.

(10) Susi, T.; Meyer, J. C.; Kotakoski, J. Quantifying Transmission Electron Microscopy Irradiation Effects Using Two-Dimensional Materials. *Nat. Rev. Phys.* **2019**, *1*, 397–405.

(11) Yoshimura, A.; Lamparski, M.; Giedt, J.; Lingerfelt, D.; Jakowski, J.; Ganesh, P.; Yu, T.; Sumpter, B. G.; Meunier, V. Quantum Theory of Electronic Excitation and Sputtering by Transmission Electron Microscopy. *Nanoscale* **2023**, *15*, 1053–1067.

(12) Neese, F. Software Update: The ORCA Program System, Version 4.0. *WIREs Comput. Mol. Sci.* **2018**, *8*, e1327.

(13) Neese, F. Software Update: The ORCA Program System—Version 5.0. *WIREs Comput. Mol. Sci.* **2022**, *12*, e1606.

(14) Neese, F. The ORCA Program System. *WIREs Comput. Mol. Sci.* **2012**, *2*, 73–78.

(15) Riley, K. E.; Op't Holt, B. T.; Merz, K. M. Critical Assessment of the Performance of Density Functional Methods for Several Atomic and Molecular Properties. *J. Chem. Theory Comput.* **2007**, *3*, 407–433.

(16) Riley, K. E.; Merz, K. M. Insights into the Strength and Origin of Halogen Bonding: The

Halobenzene−Formaldehyde Dimer. *J. Phys. Chem. A* **2007**, *111*, 1688–1694.

(17) Schultz, N. E.; Zhao, Y.; Truhlar, D. G. Databases for Transition Element Bonding: Metal−Metal Bond Energies and Bond Lengths and Their Use To Test Hybrid, Hybrid Meta, and Meta Density Functionals and Generalized Gradient Approximations. *J. Phys. Chem. A* **2005**, *109*, 4388–4403.

(18) Schultz, N. E.; Zhao, Y.; Truhlar, D. G. Density Functionals for Inorganometallic and Organometallic Chemistry. *J. Phys. Chem. A* **2005**, *109*, 11127–11143.

(19) Farmanzadeh, D.; Abdollahi, T. Investigation on the Chemical Active Sites of Copper Nanoclusters as Nanocatalyst for the Adsorption of Acetylene: Calibration of DFT Method and Basis Set. *Theor. Chem. Acc.* **2016**, *135*, 49.

(20) Yang, Y.; Weaver, M. N.; Merz, K. M. J. Assessment of the "6-31+G** + LANL2DZ" Mixed Basis Set Coupled with Density Functional Theory Methods and the Effective Core Potential: Prediction of Heats of Formation and Ionization Potentials for First-Row-Transition-Metal Complexes. *J. Phys. Chem. A* **2009**, *113*, 9843–9851.

(21) Chamberlain, T. W.; Biskupek, J.; Skowron, S. T.; Markevich, A. V; Kurasch, S.; Reimer, O.; Walker, K. E.; Rance, G. A.; Feng, X.; Müllen, K.; Turchanin, A.; Lebedeva, M. A.; Majouga, A. G.; Nenajdenko, V. G.; Kaiser, U.; Besley, E.; Khlobystov, A. N. Stop-Frame Filming and Discovery of Reactions at the Single-Molecule Level by Transmission Electron Microscopy. *ACS Nano* **2017**, *11*, 2509–2520.

(22) McKinley, W. A.; Feshbach, H. The Coulomb Scattering of Relativistic Electrons by Nuclei. *Phys. Rev.* **1948**, *74*, 1759–1763.

(23) Liang, B. Condition-Optimized AC-HRTEM Characterization of Beam-Sensitive 2D Organic Frameworks, Ulm, 2023. https://doi.org/https://doi.org/10.18725/OPARU-49443.
